# Cannabidiol inhibits neuroinflammatory responses and circuit-associated synaptic loss following damage to a songbird vocal pre-motor cortical-like region

**DOI:** 10.1038/s41598-023-34924-z

**Published:** 2023-05-16

**Authors:** Mark Tripson, Karen Litwa, Ken Soderstrom

**Affiliations:** 1 Department of Pharmacology and Toxicology, Brody School of Medicine at East Carolina University, Greenville, NC 27834 USA; 2 Department of Anatomy and Cell Biology, Brody School of Medicine, East Carolina Diabetes and Obesity Institute, East Carolina University, Greenville, NC 27834 USA

**Keywords:** Neuroscience, Learning and memory, Molecular neuroscience, Neural circuits, Synaptic plasticity, Inflammation, Pharmacology

## Abstract

The non-euphorigenic phytocannabinoid cannabidiol (CBD) has been used successfully to treat childhood-onset epilepsies. These conditions are associated with developmental delays that often include vocal learning. Zebra finch song, like language, is a complex behavior learned during a sensitive period of development. Song quality is maintained through continuous sensorimotor refinement involving circuits that control learning and production. Within the vocal motor circuit, HVC is a cortical-like region that when partially lesioned temporarily disrupts song structure. We previously found CBD (10 mg/kg/day) improves post-lesion vocal recovery. The present studies were done to begin to understand mechanisms possibly responsible for CBD vocal protection. We found CBD markedly reduced expression of inflammatory mediators and oxidative stress markers. These effects were associated with regionally-reduced expression of the microglial marker TMEM119. As microglia are key regulators of synaptic reorganization, we measured synapse densities, finding significant lesion-induced circuit-wide decreases that were largely reversed by CBD. Synaptic protection was accompanied by NRF2 activation and BDNF/ARC/ARG3.1/MSK1 expression implicating mechanisms important to song circuit node mitigation of oxidative stress and promotion of synaptic homeostasis. Our findings demonstrate that CBD promotes an array of neuroprotective processes consistent with modulation of multiple cell signaling systems, and suggest these mechanisms are important to post-lesion recovery of a complex learned behavior.

## Introduction

Cannabis and many of the bioactive molecules it produces have been studied for decades^1^. These efforts have resulted in identification of hundreds of phytocannabinoids, with two standing-out as therapeutics: cannabidiol (CBD) and Δ^9^-tetrahydrocannabinol (THC). Until recently, interest in CBD had been eclipsed by a focus on the more-dramatically effective and euphorigenic THC. More subtly-acting CBD is now receiving increased therapeutic attention as a botanically-derived formulation has been approved for marketing in the US to treat childhood seizure disorders^2^.

These seizure disorders are associated with delayed language development^3^. Caretaker surveys from childhood epilepsy trials suggest CBD improves vocal communication^4,5^. To investigate potential utility of CBD in speech and language disorders we have used a vocal learning songbird, zebra finch, as a pre-clinical animal model.

Like human language, song is learned during a sensitive period of development^6^. Also like humans, songbird vocal learning depends upon establishment of circuits linking cortical, striatal and thalamic brain regions^7^. Gene expression profiles demonstrate distinct similarities between songbird and human vocal learning^8,9^ and motor regions^10^. For example, evidence supports functional similarities between human laryngeal motor cortex (LMC) layers 2, 3 and 6 and songbird HVC (proper name), that each drive vocal motor output (from LMC layer 5 and songbird robust nucleus of the arcopallium [RA], respectively^9^).

Previously, using a bilateral HVC microlesion method to disrupt quality of vocalizations^11^ we found that daily treatments with 10 mg/kg CBD decreases the magnitude of song disruption and improves recovery time^12^. For the present study, to reduce animal impact and allow for statistically powerful within-subject controls, we adopted a unilateral lesion approach. Initial results indicated that, like the bilateral method, unilateral HVC microlesions reversibly disrupt song patterns, but with a more modest magnitude of effect. In addition, we found that left hemisphere microlesions produced greater vocal disruption than did right hemisphere microlesions, consistent with songbird vocal lateralization reported by others^13^. Note that because recovery depends upon auditory perception (deafened birds do not improve^14^) adult sensorimotor relearning is required. Our current goal is to improve understanding of mechanisms by which CBD protects vocal quality and improves learning-dependent recovery of a complex motor behavior.

**Figure 1:**
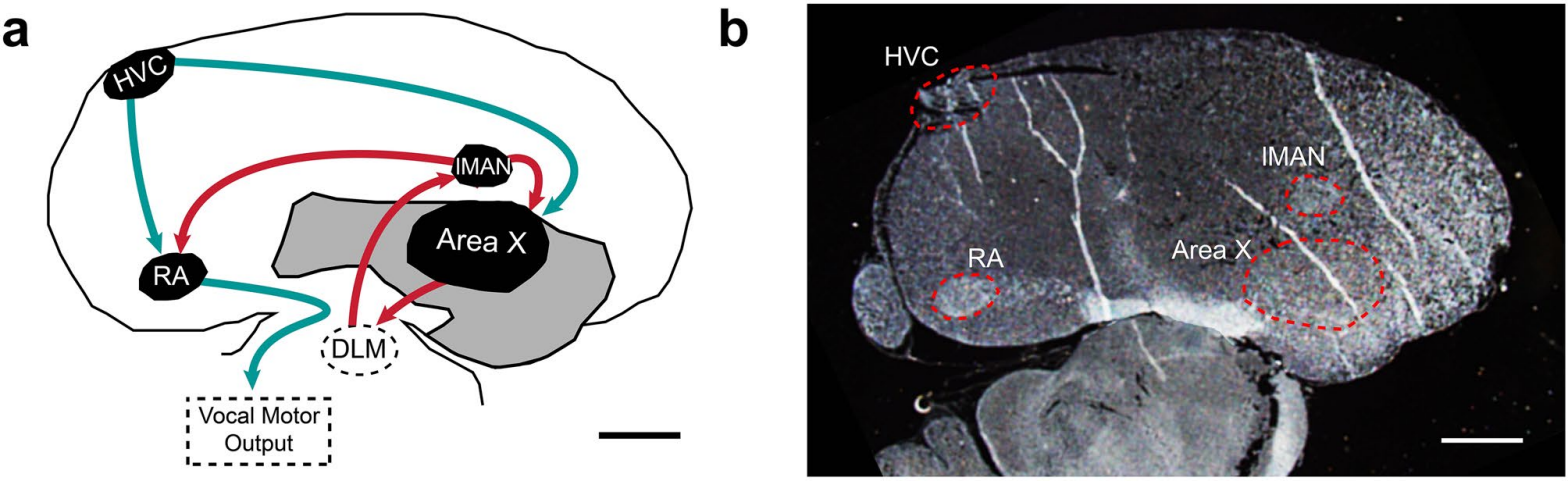
Summary of song brain regions of interest. (**A**) Schematic representation of brain regions focused upon and their interconnections. Microlesions target HVC and produce vocal deficits with recovery dependent on sensorimotor learning (deafened birds do not recover^14^). Red arrows represent the anterior forebrain pathway (AFP), a cortico-basal ganglia-thalamic circuit critical for vocal learning and inducing vocal variability through adulthood. Green arrows represent vocal motor pathways. Dashed regions indicate approximate x,y locations of regions not visible in panel B. (**B**) Is a representative darkfield image used to identify sections with regions of interest and to define their borders for superimposing on images obtained later via confocal microscopy. This image was used to produce the camera lucida-type tracing in Panel A. Note the distinct nuclear organization of song regions contrasts with laminated mammalian cortex and allows targeting for lesioning, dissection, and other manipulation. Rostral is approximately right, dorsal up, and bars = 1 mm. Abbreviations: HVC (proper name), lMAN (lateral magnocellular nucleus of the anterior nidopallium), RA (robust nucleus of the arcopallium), Area X (Area X of striatum, note that songbird striatum also contains pallidal projection neurons and interneurons that anatomically distinguish it from mammalian striatum that separates these features^20^).

Accumulating evidence shows that CBD has anti-inflammatory and anti-oxidative stress activity involving immune cell activation and migration to areas of CNS injury^15^. For example, a gene expression study using microarray-based gene profiling indicates CBD attenuates several cellular events through modulating the expression of pro-inflammatory transcription factors, cytokines and cytokine receptors (that are notably released by microglia and controlled by the master anti-oxidant regulator, NRF2^16,17^). More recent RNASeq experiments demonstrate CBD-altered expression of immune mediators in prefrontal cortex and likely related cognitive improvement in a rat schizophrenia model^18^. In an Alzheimer’s mouse model CBD stimulates hippocampal anti-inflammatory and promotes homeostatic autophagy gene expression^19^. Given these examples of anti-inflammatory CBD activity, we suspected involvement of similar mechanisms in vocal recovery. To begin to test this possibility, we investigated CBD modulation of post-microlesion expression of inflammatory cytokines, markers of neuronal oxidative stress, microglia/macrophage infiltration and related changes in synaptic densities within relevant song control regions (Fig. 1).

## Results

### CBD reduces markers of inflammation and oxidative stress following HVC microlesions

**Figure 2:**
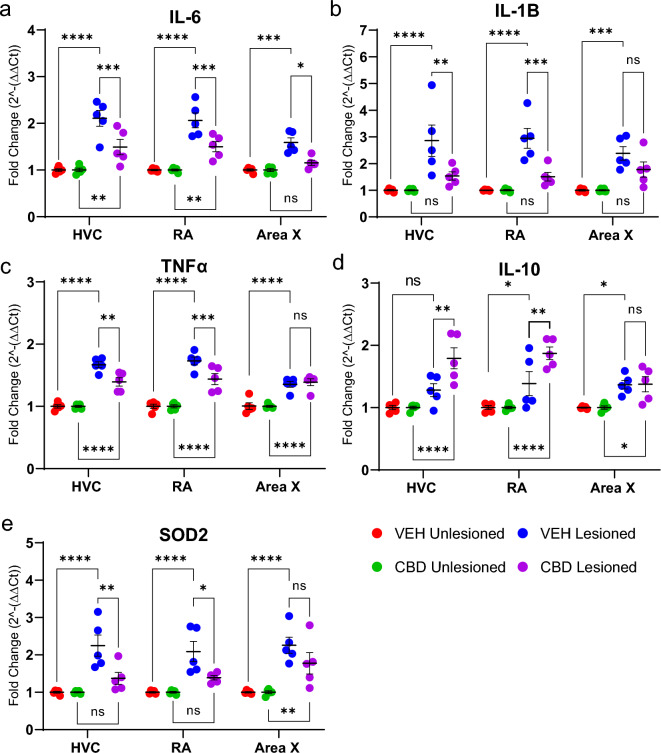
CBD promotes a pattern of anti-inflammatory and anti-oxidative stress-related gene expression. Brain regions from both vocal-motor (HVC and RA) and -learning circuits (Area X) were micropunch dissected, total RNA extracted, cDNA synthesized, and PCR amplified. Gene expression of IL-1B, IL-6, IL-10, TNFα, and SOD2 were normalized to the endogenous control (GAPDH) and fold change from the unlesioned hemisphere was expressed as 2^−∆∆CT^. To quantify, cDNA from n = 5 per group was amplified in triplicate and the average cycle threshold (Ct) value was calculated. The mean Ct value was then used for further analysis. (**A**) CBD significantly decreased mean fold expression of pro-inflammatory IL-6 in HVC, RA, and Area X. (**B**) CBD decreased mean fold expression of IL-1Β in HVC and RA but not Area X. (**C**) TNFα was decreased in HVC and RA but not Area X. (**D**) mean fold expression of the anti-inflammatory mediator IL-10 was increased in HVC and RA but not significantly in Area X. (**E**) Expression of the marker of oxidative stress, superoxide dismutase 2 (SOD2) was decreased within HVC and RA. Note that tissue was obtained 24 h post-lesion and thus represents a “snap-shot” of inflammatory cytokine expression that is known to vary with time post-injury^24^. Group differences were assessed by mixed-models ANOVA with Sidak’s multiple comparison correction.

In other systems CBD exhibits anti-inflammatory and antioxidant efficacy that is in part responsible for its neuroprotective activity^21,22^. To evaluate whether CBD-improved vocal recovery may involve similar anti-inflammatory efficacy, we quantified expression of several pro- and anti-inflammatory mediators (Fig. 2A–D). Using the micro punch dissection technique (described below) 24 h after microlesions we isolated tissue for RNA extraction, cDNA synthesis and amplification from song regions within nodes of motor (HVC & RA) and learning essential circuits (Area X, note the micro punch dissection approach used is illustrated in Supplemental Fig. S2). Mixed models ANOVA revealed CBD treatments significantly reduced fold expression (relative to vehicle controls) of the pro-inflammatory mediators IL-6 (within HVC by mean = 0.615 [0.243–0.987], *p* = 0.0002; IL-1Β (within HVC by mean = 1.33 [0.427–2.222], *p* = 0.0015; and RA by mean = 1.44 [0.542–2.337], *p* = 0.0005; RA by mean = 0.563 [0.192–0.935], *p* = 0.0008; and Area X by mean = 0.435 [0.063–0.807], *p* = 0.0141, see Fig. 2A–C). The lesioned area, HVC and RA showed additional reduction of TNFα expression (HVC by mean 0.275 [0.087–0.462], *p* = 0.0016, RA by mean = 0.292 [0.105–0.48], *p* = 0.0008), that was not statistically significant in Area X (by mean = 0.035 [− 0.222–0.153], *p* = 0.960). In addition to decreasing expression of pro-inflammatory mediators, CBD has also been shown to upregulate the anti-inflammatory cytokine IL-10^23^. We quantified differences in IL-10 expression and found them to be significantly increased in HVC of CBD-treated birds (by mean = 0.512 [0.152–0.872, *p* = 0.0023) and RA (by mean = 0.487 [0.127–0.846], *p* = 0.004) but not in Area X (by mean = 0.008 [− 0.368–0.352], *p* > 0.99, see Fig. 2D).

Consistent with anti-inflammatory efficacy, CBD has been shown to affect redox balance through both direct and indirect antioxidant activity^25^. Expression of superoxide dismutase 2 (SOD2), a gene that encodes the mitochondrial protein SOD2 responsible for binding of superoxide byproducts, showed a significant CBD-related decrease within HVC (by mean fold = 0.8768 [0.269–1.49], *p* = 0.002), and RA (mean fold = 0.701 [0.093–1.31], *p* = 0.018), but not in Area X (mean fold = 0.4841 [− 0.124–1.092], *p* = 0.162, see Fig. 2E).

**Figure 3:**
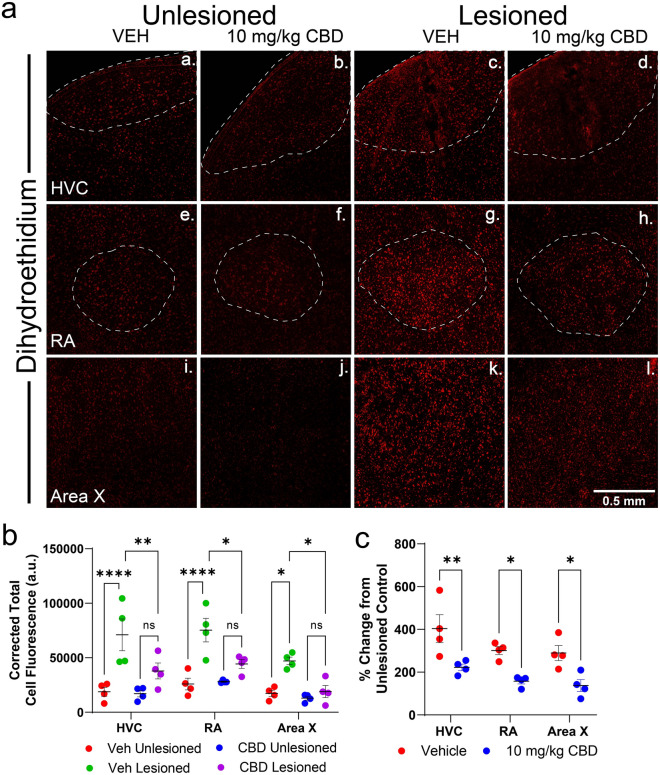
CBD decreased reactive oxygen species in lesioned hemispheres as indicated by intensity of dihydroethidium staining (DHE, in red). (**A**) Representative confocal images displaying regional DHE staining indicative of reactive oxygen species (ROS) as a function of lesion condition and treatment. (**B**) Summary of mean Corrected Total Cell Fluorescence (CTCF) of DHE staining within each area using a circular area with a diameter of 0.5 mm (see methods for CTCF calculation details). In the vehicle group, microlesions significantly increased total fluorescence of DHE staining within HVC, RA and Area X. Significantly increased lesion-induced DHE staining was not observed in CBD treated groups. In all brain regions of lesioned hemispheres, DHE staining was significantly lower in CBD-treated groups compared to controls. (**C**) Comparing % control transformed measures of DHE staining within lesioned hemispheres of vehicle- and CBD-treated animals, CBD reduced intensities within HVC, RA, and Area X. CBD treatment significantly reduced lesion related changes in DHE expression in all regions as a percentage of their respective control hemisphere. Mean grey values were determined and corrected for background fluorescence using the average intensity outside of each song region studied. Comparisons were made and significance determined using Sidak’s correction for multiple comparisons following mixed-models ANOVA (n = 4 per group).

To confirm that decreased SOD2 expression was accompanied by a general decrease in oxidative stress, we used the superoxide indicator dihydroethidium (DHE, see Fig. 3). When oxidized, DHE intercalates double-stranded genomic DNA marking nuclei with red fluorescence. In the vehicle group we found microlesions significantly increased corrected total cell fluorescence (CTCF) of DHE staining within HVC (by mean = 52,215 [26,635–77,796], *p* < 0.0001), RA (by 49,460 [23,880–75,041], *p* < 0.0001), and Area X (by 29,817 [4237–55,397], *p* = 0.0152 see Fig. 3A). In contrast, there were no significant differences in DHE staining between the unlesioned hemispheres of vehicle- and CBD-treated animals (Fig. 3B VEH Unlesioned vs CBD Unlesioned 3a,b,e,f, I,j). (HVC, by mean = 1777 [23,803–27,358], *p* > 0.9999; RA, by mean = 2194 [− 27,775–23386], *p* > 0.9999; and Area X, by mean = 4280 [− 21,300–29,861], *p* = 0.9980). Comparing DHE staining within lesioned hemispheres of vehicle- and CBD-treated animals, CBD reduced intensities within HVC (by mean = 33,153 [7573–58,734], *p* = 0.0056), RA (by mean = 30,998 [5417–56,578], *p* = 0.0107), and Area X (by mean = 28,089 [2508–53,669], *p* = 0.0250 see Fig. 3B). Additionally, when comparing treatment groups to their respective unlesioned controls, CBD treatments significantly reduced lesion related changes in DHE expression in all regions (HVC by mean = 181.5% [53.91–309.1], *p* = 0.0045; RA by mean = 143.6% [16.04–271.2], *p* = 0.0248; and Area X by mean = 152.0% [24.40–279.6], *p* = 0.0171 see Fig. 3C).

**Figure 4:**
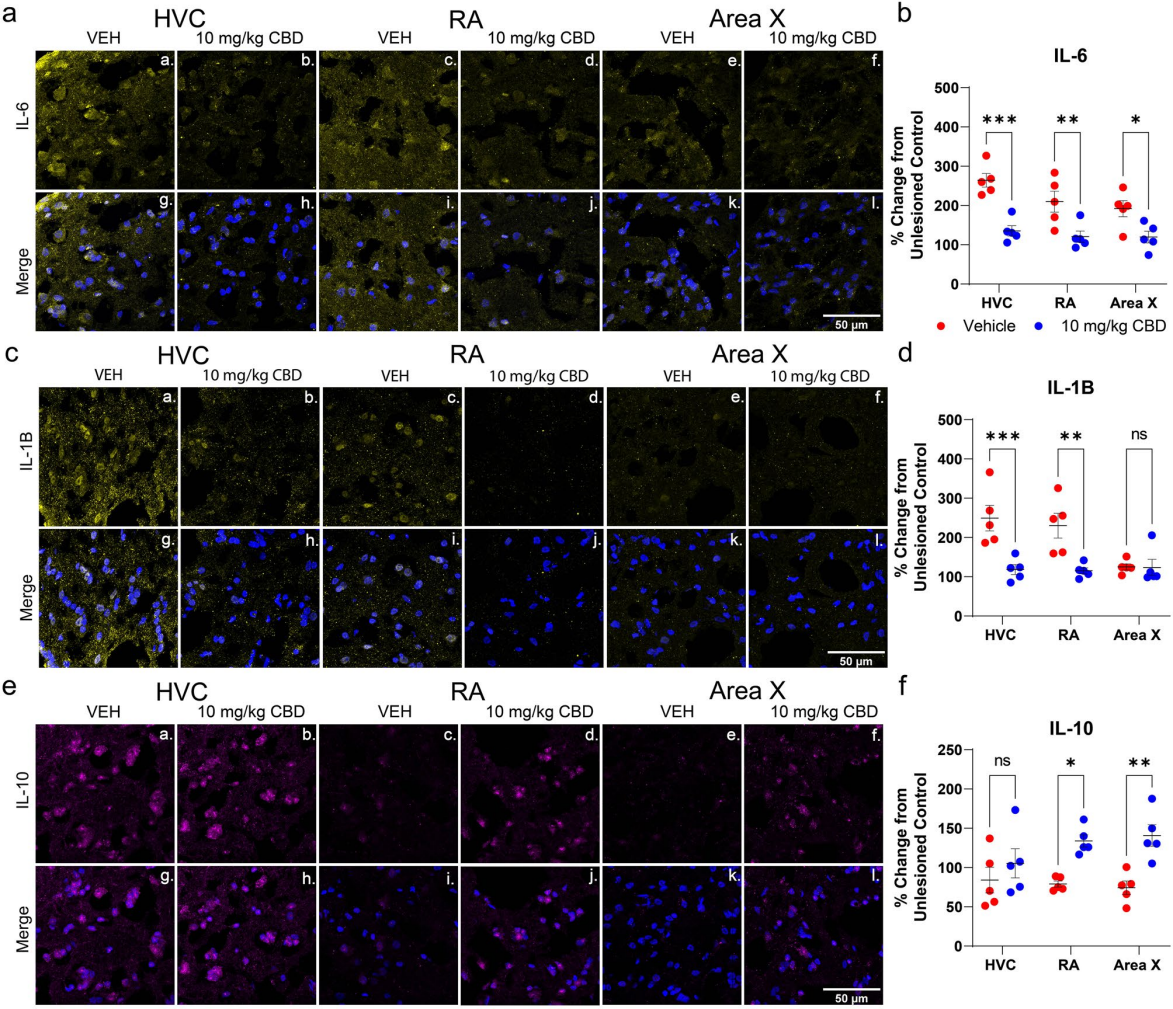
CBD alters inflammation-related cytokine densities. Daily treatments with 10 mg/kg were associated with modulation of IL-1Β, IL-6 and IL-10 protein expression 24 h after unilateral HVC lesions. Shown are representative immunofluorescent confocal images of antibody staining targeting IL-1Β, IL-6 and IL-10 within motor (HVC & RA) and learning-essential (Area X) regions. **(Aa-Al)**, lesioned hemisphere images showing representative regional distribution of IL-6 in vehicle- vs. CBD-treated birds. **(Ca–Cl)**, images demonstrating regional distribution of IL-1Β. **(Ea-El)**, confocal images demonstrating regional distribution of IL-10. Representative images for the unlesioned hemisphere are presented in Supplemental Fig. S3. B, IL-6 fluorescence as percent change from unlesioned control hemisphere by song region and drug treatment. CBD significantly reduced IL-6 expression in HVC, RA, and Area X. D, IL-1B fluorescence as percent change from unlesioned control hemisphere by song region and drug treatment. CBD significantly decreased IL-1Β intensity in HVC, and RA. F, anti-inflammatory IL-10 fluorescence as percent change from unlesioned control hemisphere by song region and drug treatment. CBD significantly increased IL-10 intensity in RA and Area X. Differences were determined by mixed-models ANOVA followed by Sidak-corrected post-hoc comparisons. Image J software was used to analyze z-stack images projected at maximum intensity and threshold was applied consistently across all images for n = 5 per group.

To confirm that CBD-induced anti-inflammatory and anti-oxidative patterns of gene expression result in functional protein level changes we used immunofluorescence approaches. We measured relative immunofluorescence using antibodies targeting IL-6, IL-1B and IL-10 and expressed results as a percentage of unlesioned hemispheres (Fig. 4A–F, note that selectivity of antibodies used is supported by staining of single predominant bands of expected size: see Supplemental Fig. S6). CBD significantly reduced lesion-related IL-6 protein expression in HVC (by mean percent = 128.9% [62.32–195.4], *p* = 0.0001), and RA (by 89.37% [22.84–155.9], *p* = 0.0063 Fig. 4A,B). For IL-1Β, CBD treatment significantly decreased expression in HVC (by mean = 131.2% [53.55–208.8], *p* = 0.0007), and RA (by mean = 115.0% [37.36–192.6], *p* = 0.0026, Fig. 4C,D). Expression of IL-6, but not IL-1B, was differentially regulated in Area X (IL-6 by 72.21% [5.679–138.7], *p* = 0.031; and IL-1B by 1.44% [− 76.17–79.04], *p* > 0.9999, Fig. 4B,D). CBD significantly increased anti-inflammatory IL-10 in RA (by mean = 54.9% [9.388–100.4], *p* = 0.0147) and Area X (by mean = 66.5% [20.96–111.9], *p* = 0.0030) while there was no statistical difference in the lesioned site (HVC by mean = 21.4% [− 66.85–24.14], *p* = 0.5612 Fig. 4E,F).

**Figure 5:**
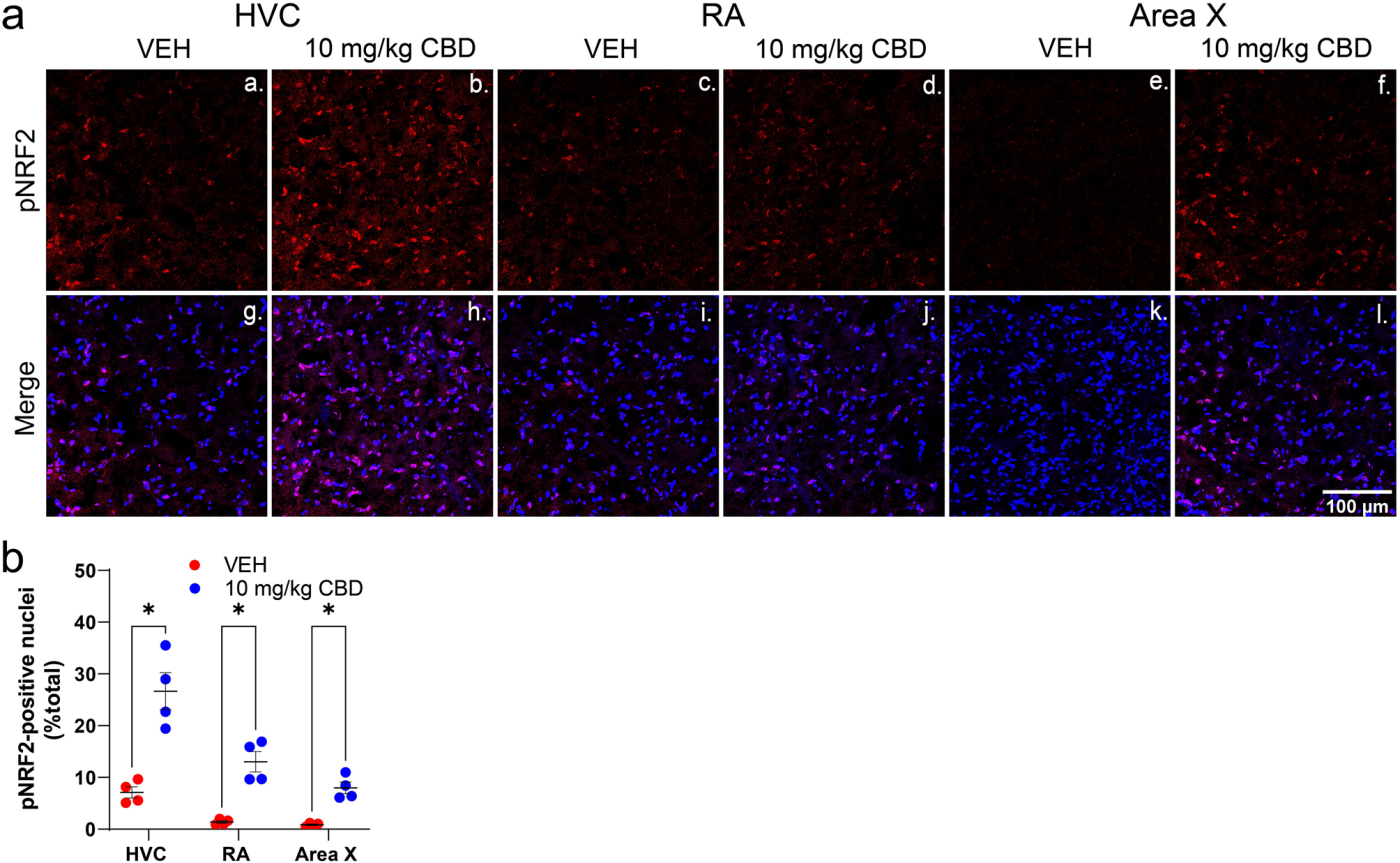
CBD increases nuclear localization of the antioxidant response-regulating transcription factor pNRF2. **(A)**, Images within song regions were taken from tissue collected 24 h after unilateral HVC lesions. Top row **(a-f)** is pNRF2 staining. Bottom row **(g-l)** is pNRF2 staining in red merged with that of Hoechst nuclear staining in blue. **(B)**, pNRF2 nuclear staining in song regions of interest expressed as a percentage of total nuclear staining. Results indicate CBD treatment significantly increased nuclear pNRF2 within HVC, RA and Area X consistent with antioxidant responses. Image J software was used to analyze z-stack images projected at maximum intensity and threshold was applied consistently across all images. Group differences were determined using mixed-models ANOVA followed by Sidak-corrected post-hoc comparisons for n = 4 per group.

The anti-inflammatory and anti-oxidative activity of CBD suggests involvement of NRF2-mediated regulation of redox, mitochondrial and inflammatory processes to maintain homeostasis. Results indicate CBD treatment significantly increased nuclear levels of pNRF2 within all regions of interest: in HVC (by mean percent = 19.6 [3.7–35.4], *p* = 0.0259), RA (by mean = 11.65 [2.39–20.9], *p* = 0.0262) and Area X (by mean = 7.1 [1.8–12.43], *p* = 0.0218, Fig. 5B).

### CBD effectively reduces expression of the microglial marker TMEM119

**Figure 6:**
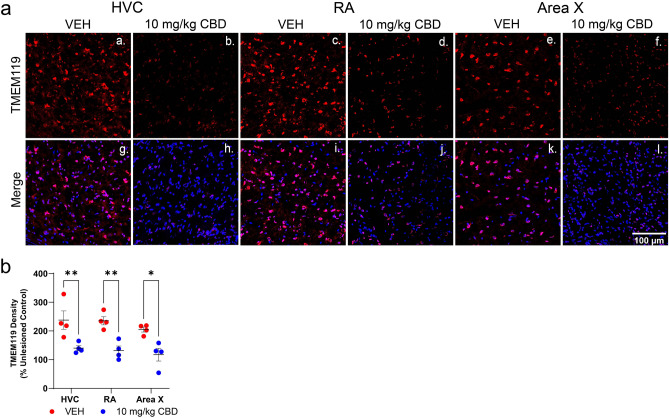
CBD treatments decrease density of the microglial marker TMEM119 within song regions of lesioned hemispheres. (aa-af), TMEM119 immunofluorescence marks microglia, in which a high density of fluorescence is present in vehicle-treated HVC, RA and Area X. Lower TMEM119 staining is evident in the CBD treated group. (ag-al), Merge images of TMEM119 and Hoechst-stained nuclei. (**b**) TMEM119 density expressed as TMEM119 mean grey value relative Hoechst staining as percentage of the unlesioned hemisphere. Within HVC, RA and Area X, TMEM119 densities were significantly lower in CBD-treated animals relative to vehicle controls. Group differences were determined using mixed-models ANOVA followed by Sidak corrected post-hoc comparisons for n = 4 per group.

Microglia activation, recruitment, and phagocytosis are primary inflammatory responses after injury, known to be mediated by the release of cytokines and superoxide production with downstream effects on the complement cascade^26,27^. Previous evidence links microglial activation with transient elevation of pro-inflammatory genes (i.e. IL-1Β & IL-6) that have been observed at peak neuronal death during tissue damage^27^. Given our results showing increased expression of pro-inflammatory cytokines 24 h after microlesion surgeries, we explored the possibility of microglial involvement as part of CBD’s mechanism of action in our system. Using TMEM119 as a microglia marker, we studied lesion-induced TMEM119 staining within song regions (HVC, RA, and Area X) and CBD effects on this expression (Fig. 6A). Results indicate within HVC, RA and Area X, TMEM119 levels were significantly lower in CBD-treated animals relative to controls (by mean 97.61% [27.59–167.6], *p* = 0.0053; 103.8% [33.77–173.8], *p* = 0.0031 and; by 87.39% [17.37–157.4], *p* = 0.0123, respectively (Fig. 6B). Note that our present focus on microglia represents a first step, and does not preclude likely involvement of other cell types in microlesion-induced inflammatory responses (e.g. reactive astrocytes)^28^.

### CBD inhibition of lesion-induced synaptic loss

**Figure 7:**
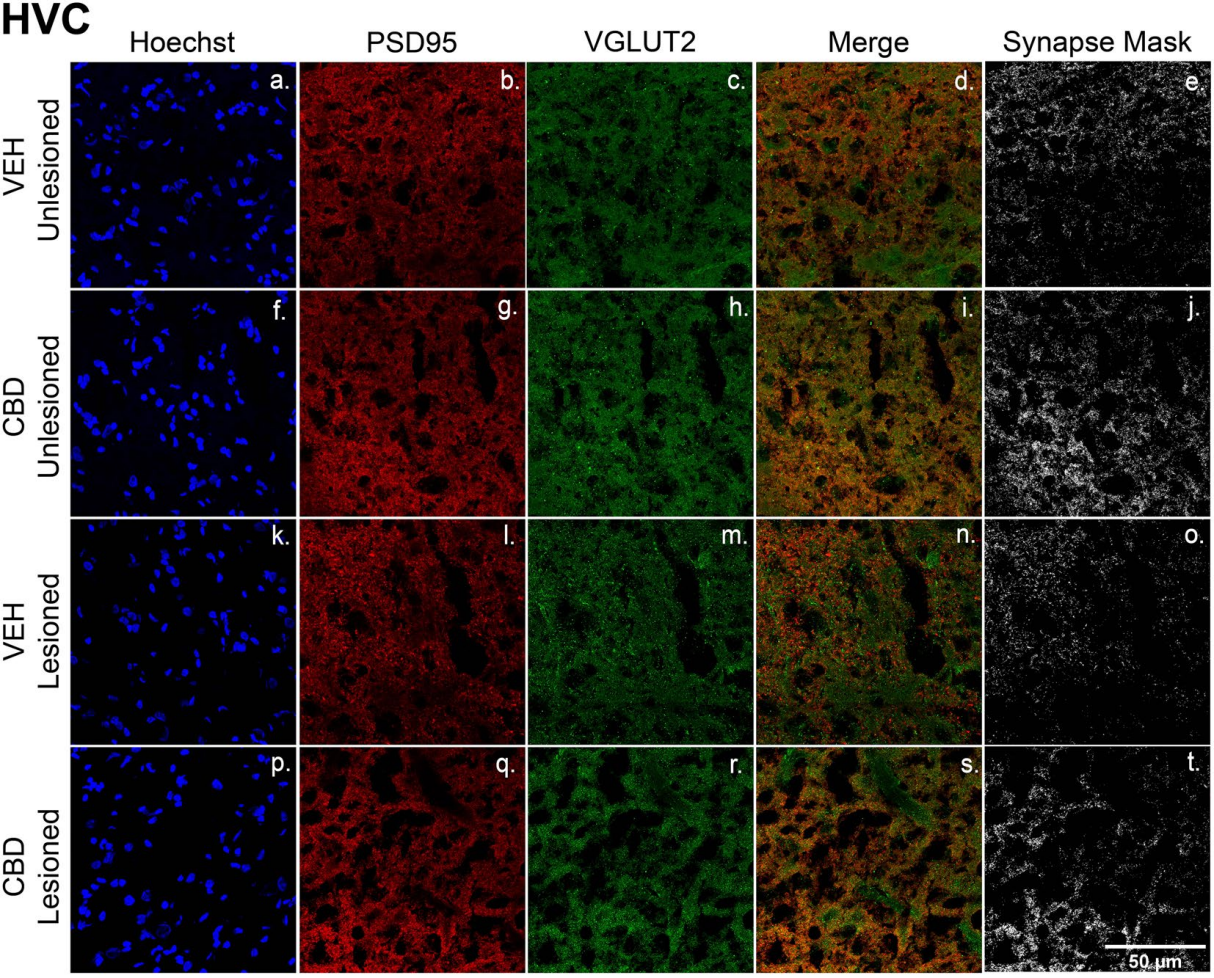
CBD treatments protect glutamatergic synaptic densities from lesion-related losses within HVC. (**a**–**t**) Representative confocal images of immunofluorescence illustrating synaptic density in four groups: VEH Unleisoned (**a**–**e**), CBD Unlesioned (**f**–**j**), VEH Lesioned (**k**–**o**), and CBD lesioned (**p**–**t**). Stains are divided into columns with Hoechst, PSD95 (postsynaptic marker), and VGLUT2 (presynaptic marker) respectfully. Column four is a merge of PSD95 and VGLUT2 and column 5 shows a mask of the colocalized puncta. The synapse masks show that relative to VEH controls CBD groups have significantly higher glutamatergic synapse densities 24 h post-lesions.

**Figure 8:**
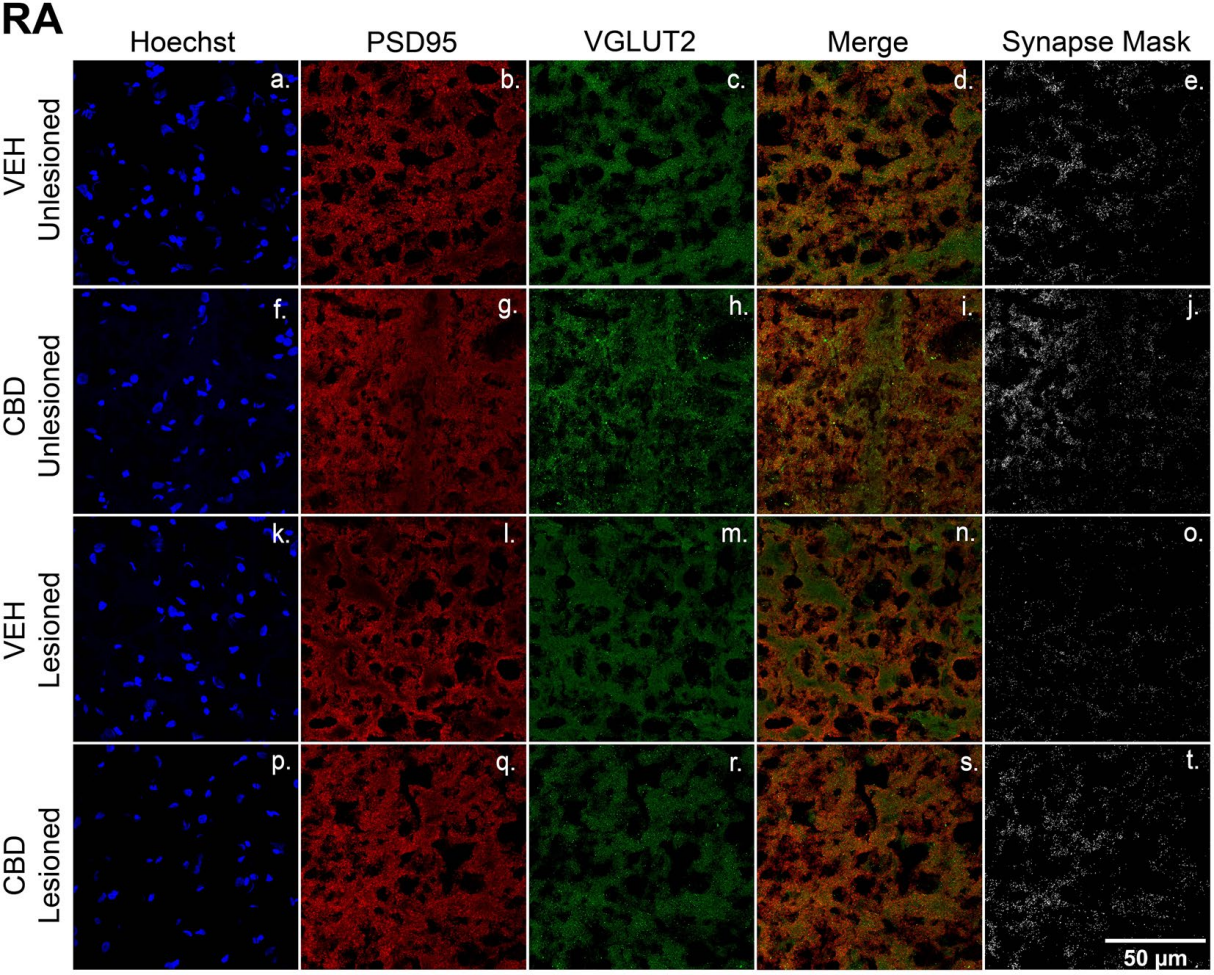
CBD treatments protect glutamatergic synaptic densities from lesion-related losses within RA. (**a**–**t**) Representative confocal images of immunofluorescence illustrating synaptic density in four groups: VEH Unleisoned (**a**–**e**), CBD Unlesioned (**f**–**j**), VEH Lesioned (**k**–**o**), and CBD Lesioned (**p**–**t**). Stain is divided into columns with Hoechst, PSD95 (postsynaptic marker), and VGLUT2 (presynaptic marker) respectfully. Column four is a merge of the two stains and column 5 shows a mask of the colocalized puncta of PSD95 and VGLUT2. The synapse masks show that relative to VEH controls CBD groups have significantly higher glutamatergic synapse densities 24 h postlesions.

**Figure 9:**
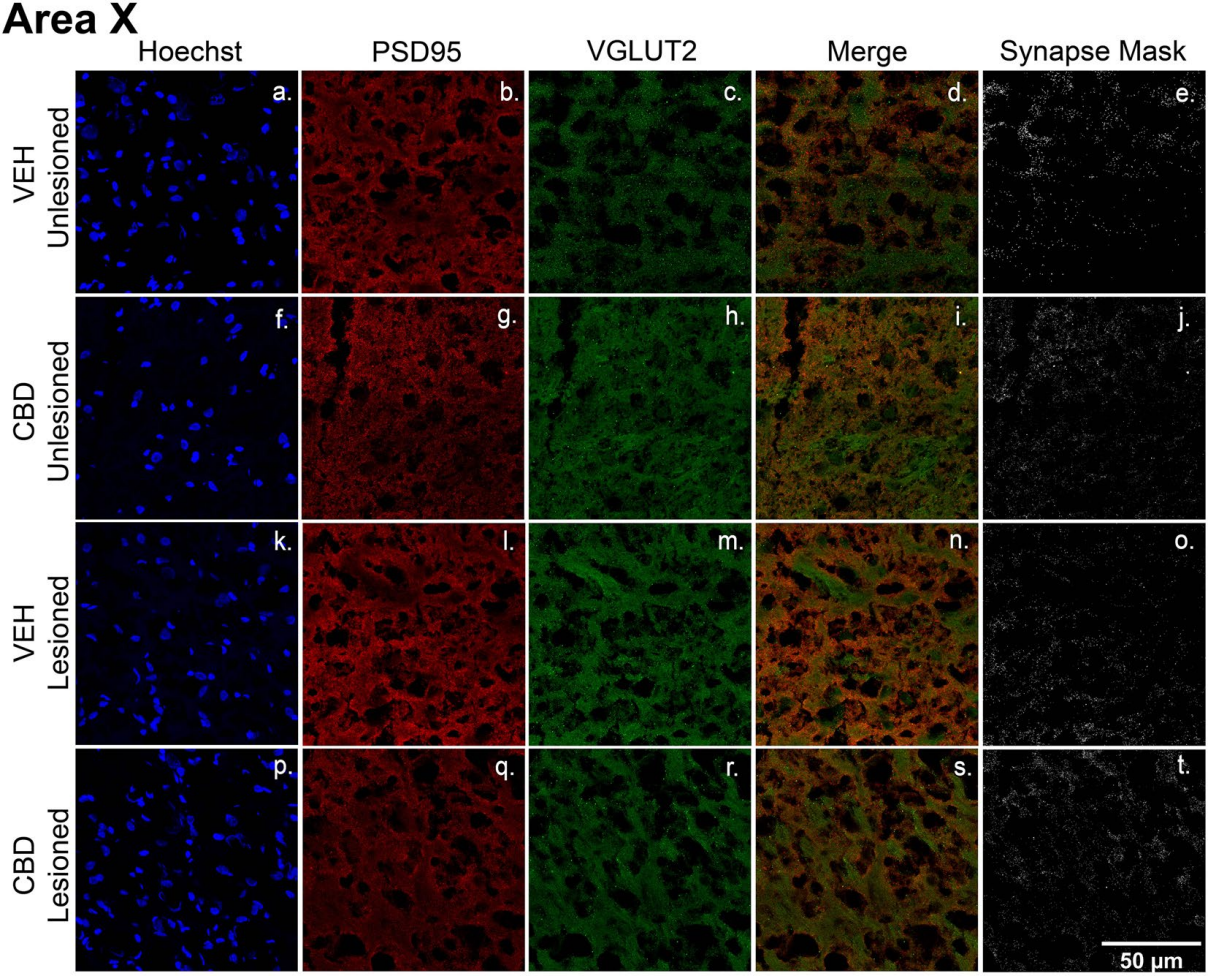
CBD treatments protect glutamatergic synaptic densities from lesion-related losses within Area X. A-T, Representative confocal images of immunofluorescence illustrating synaptic density in four groups: VEH Unleisoned (**a**–**e**), CBD Unlesioned (**f**–**j**), VEH Lesioned (**k**–**o**), and CBD Lesioned (**p**–**t**). Stain is divided into columns with Hoechst, PSD95 (postsynaptic marker), and VGLUT2 (presynaptic marker) respectfully. Column 4 is a merge of the two stains and column 5 shows a mask of the colocalized puncta of PSD95 and VGLUT2. The synapse masks show that relative to VEH controls CBD groups have significantly higher glutamatergic synapse densities 24 h post-lesions.

**Figure 10:**
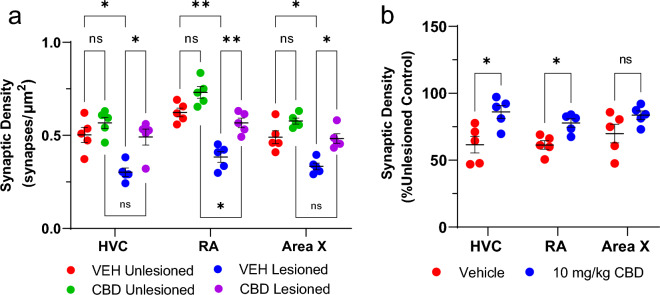
CBD treatments protect glutamatergic synaptic densities from lesion-related losses. (**a**) Quantification of song region synaptic densities within unlesioned and lesioned hemispheres of vehicle- and CBD-treated songbirds. In the vehicle group, unilateral microlesions decreased the synaptic density in all three regions examined (HVC, RA, and Area X). Conversely, in the CBD group there was no significant lesion effects in HVC or Area X. This protection was not as robust in RA, although the deficit was decreased. CBD treatment had a profound effect on the post lesion synaptic density compared to post lesion densities of vehicle treated birds in all three regions of interest. (**b**) Lesion related change in synaptic density expressed as colocalized puncta transformed to percentage of the unlesioned control hemisphere. Within HVC and RA, CBD groups had a significant increase in post-lesion synaptic density while Area X did not differ significantly. This indicates a significant protection of synapses in key areas of vocal production. For analysis, each z-stack image set was post-processed and projected at maximum intensity. Puncta of colocalized VGLUT-2 and PSD-95 within each region were defined for particle analysis with threshold applied from n = 5 animals per group. Glutamatergic synapse densities were then quantified as percent change from the unlesioned control hemisphere. Significance was assessed and appropriate comparisons made using mixed-models ANOVA with Sidak’s correction for multiple comparisons.

A critical microglial function includes phagocytotic clearing of axonal and synaptic debris following neuronal degeneration^29^. Evidence of CBD-related decreased microglial TMEM119 expression led us to question whether treatments also protected synaptic densities. To measure this, we compared colocalization of the pre- and post-synaptic markers, VGLUT2 and PSD-95 across treatment groups (Figs. 7, 8, 9, 10). We saw significant lesion-related decreases in synaptic densities. In the vehicle group, unilateral microlesions decreased the synaptic density within HVC (Fig. 10, Veh Unlesioned vs Veh Lesioned) by mean = 0.20/µm^2^ [0.025–0.38], *p* = 0.0272), RA by mean = 0.24/µm^2^ [0.11–0.37, *p* = 0.0014) and Area X by mean = 0.16/µm^2^ [0.007–0.31], p = 0.0410). Interestingly, within RA of CBD groups there was significant decrease in synaptic densities after the lesion (CBD Unlesioned vs CBD Lesioned by mean = 0.16/µm^2^ [0.022–0.30], p = 0.0234) while HVC and Area X changes were insignificant. However, CBD treatment had a profound effect on post lesion synaptic density compared to vehicle in HVC (Veh Lesioned vs CBD Lesioned, by mean = 0.19/µm^2^ [0.002–0.38], p = 0.0476) in RA (by mean = 0.18/µm^2^ [0.05–0.32], *p* = 0.0088, and Area X (by mean = 0.15/µm^2^ [0.04–0.26], *p* = 0.0122). CBD treatment also appeared to reverse lesion related decreases within HVC (CBD Unlesioned vs CBD Lesioned, mean = 0.076/µm^2^ [− 0.1126–0.2654], *p* = 0.7154 and Area X (by mean = 0.094/µm^2^ [− 0.019–0.21], p = 0.1094) but decreases remained significant in RA (mean = 0.16/µm^2^ [0.022–0.30], *p* = 0.0234). We then quantified the difference in synaptic density from the unlesioned to lesioned hemisphere as the number of colocalized puncta as a percentage of the unlesioned control hemisphere (Fig. 10B). Within HVC and RA, CBD groups had a significant increase in post-lesion synaptic density (HVC by mean = 24.4% [0.4095–48.41], p = 0.0.0464; RA by mean = 16.5% [3.019–29.90], p = 0.0186) while Area X did not differ significantly (by mean = 13.77% [12.02–39.56], p = 0.3336). Although not found to be significant, it may be important that CBD treatment tended to increase synaptic densities in unlesioned hemispheres relative to vehicle, suggesting the promotion of synaptogenesis in addition to lesion protection (Fig. 10A).

### CBD reverses microlesion-related decreased expression of synaptic scaling-related markers MSK1, Arc/Arg3.1 and BDNF

**Figure 11:**
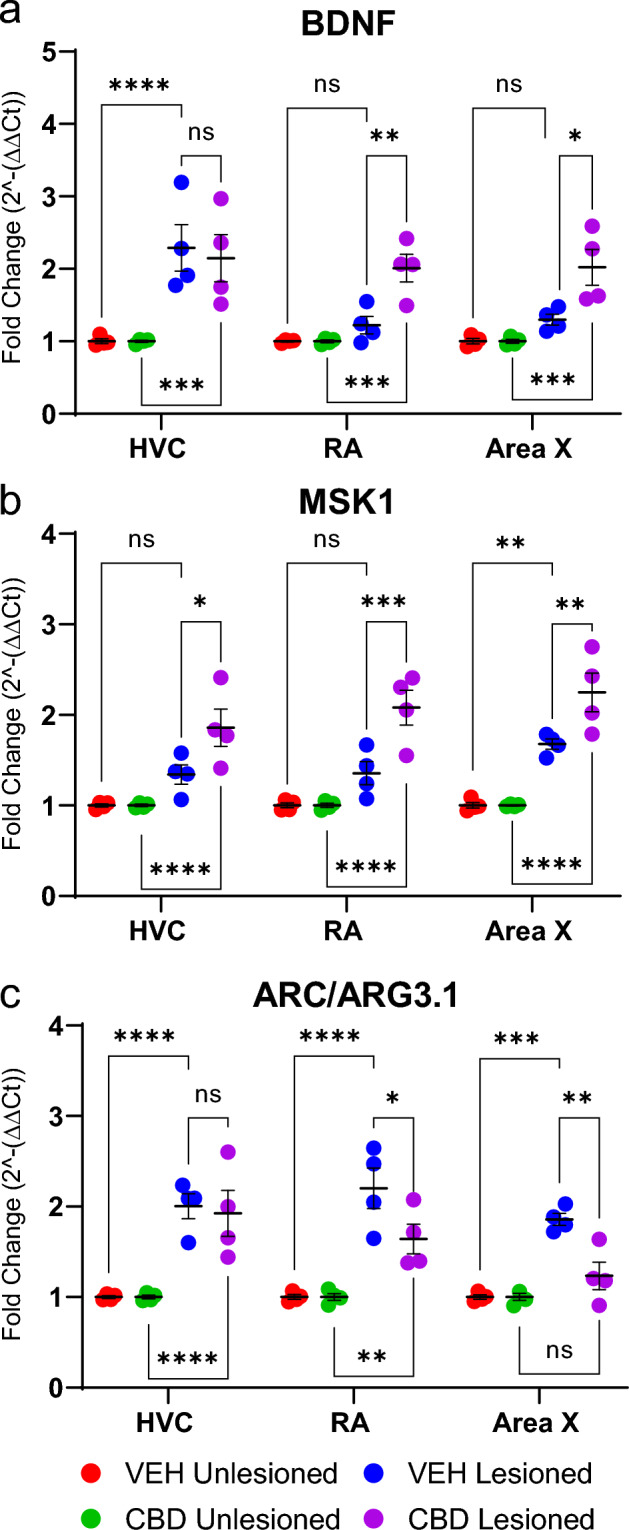
CBD influences expression of synaptic scaling regulators. Y-axes = mRNA expression of BDNF, ARC/ARG3.1, and MSK1 normalized to the housekeeping control (GAPDH) and expressed as fold change from unlesioned hemispheres (2^−∆∆CT^). The cDNA samples synthesized from groups of n = 4 subjects were amplified in triplicate and means plotted. (**a**) In RA and Area X of lesioned hemispheres, CBD significantly increased mean fold expression of BDNF relative to VEH controls. (**b**) In HVC, RA and Area X of lesioned hemispheres CBD treatment increased mean fold expression of MSK1 over VEH. (**c**) In RA and Area X of lesioned hemispheres, CBD suppressed ARC/ARG3.1 expression versus VEH. Group differences were assessed by mixed-models ANOVA with Sidak’s multiple comparison correction.

Zebra finch vocal recovery requires sensorimotor feedback^14^ which led us to investigate factors linking experience with neuroplasticity and homeostatic synaptic scaling^30^. By quantifying expression of key plasticity-related genes via qRT-PCR we found significant treatment group differences in expression of BDNF (F[3, 36] = 28.79, *p* < 0.0001), MSK1 (F[3, 36] = 57.63, *p* < 0.0001) and ARC/ARG3.1 (F[3, 36]  = 46.53, *p* < 0.0001, Fig. 11).

Post-hoc comparisons revealed that in VEH controls lesions significantly increased BDNF expression in HVC (by 1.29 fold, 95% CI = 0.66—1.92, *p* < 0.0001) but not RA or Area X (Fig. 11A). This appears related to stimulation of BDNF expression in the lesioned region, HVC, that did not extend to its projection targets, RA and Area X. This wasn’t true in CBD-treated finches where significant differences between unlesioned and lesioned-group birds were observed in each brain region (in HVC by 1.15, 95% CI = 0.52–1.78, *p* < 0.0001; in RA by 1.01, 95% CI = 0.38–1.64, *p* = 0.0007; in Area X by 1.02, 95% CI = 0.39–1.36, *p* = 0.0006).

Because evidence suggests BDNF increases MSK1 expression in experience-related synaptic plasticity^31^ we investigated potential CBD regulation of this kinase’s expression. Although lesions tended to increase MSK1 expression in VEH controls, this was only significantly different in Area X (VEH Unlesioned vs. VEH Lesioned by 0.68, 95% CI = 0.24–1.11, *p* = 0.0010, Fig. 11B). In contrast, CBD-treated birds had significantly increased MSK1 expression in each brain region of interest (in HVC by 1.25, 95% CI = 0.81–1.66, *p* < 0.0001; in RA by 1.08, 95% CI = 0.64–1.52, *p* < 0.0001; in Area X by 1.01, 95% CI = 0.38–1.64, *p* < 0.0001).

In models of homeostatic synaptic scaling, high levels of synaptic activity increase signaling of the BDNF/MSK1 pathway^32^ promoting ARC/ARG3.1 expression. ARC/ARG3.1 activity promotes internalization of excitatory AMPA receptors, decreasing excitatory synaptic strength. To test whether this ARC/ARG3.1 signaling pathway is affected by CBD treatment in our system we measured treatment effects on ARC/ARG3.1 expression. Interestingly, in each brain region of interest of vehicle-treated controls, lesions significantly increased ARC/ARG3.1 expression (in HVC by 0.08, 95% CI = − 0.40–0.56, *p* = 0.970; in RA by 0.56, 95% CI = 0.079–1.04, *p* = 0.017; in Area X by 0.62, 95% CI = 0.14—1.11, *p* = 0.0068, Fig. 11C). Comparing vehicle- and CBD-treated lesioned birds, expression of ARC/ARG3.1 was significantly lower in RA and Area X, but not HVC, of CBD-treated finches (in RA by 0.56, 95% CI = 0.08—1.04, *p* = 0.017 and; Area X by 0.62, 95% CI = 0.14–1.11, *p* = 0.007). If, as in other systems, ARC/ARG3.1 acts to internalize AMPA receptors, we would expect higher densities following CBD treatments. We are currently investigating this possibility.

## Discussion

These experiments were done to identify candidate mechanisms that could contribute to previously observed CBD acceleration of vocal recovery after HVC microlesions; and to begin to identify learning and motor regions involved. The value of studying CBD neuroprotection in songbirds lies in ability to identify processes modified within discrete nodes of circuits controlling vocal learning and production. Vocal control pathways in both songbirds and humans share convergent functional similarities that increase translational relevance relative to non-vocal learning species^9,10^. A distinct difference between avian and human vocal pathways is nuclear rather than laminated organization of pallial vocal control regions. This difference imparts advantages in targeting regions for manipulation and spatial assessment; a feature we have taken advantage of. In addition to HVC (the pre-motor cortical-like microlesion target) we have focused on its projection targets: RA (motor cortical-like) and; Area X (a learning-essential striatal region that also incorporates pallidal projection neurons). Because song recovery depends upon auditory perception-dependent sensorimotor integration (deafened birds don’t improve^14^) our system uniquely models adult loss and learning-dependent recovery of a complex motor skill.

### CBD anti-neuroinflammatory activity

Results demonstrate that in the HVC microlesion model that CBD has powerful anti-neuroinflammatory effects consistent with those well-characterized in mammalian systems^16^ (see Figs. 2 and 4). This is important as neuroinflammation is an etiological factor in development of a spectrum of CNS disorders^33^. An example is chronic neuroinflammation triggered by post-traumatic brain injury that increases likelihood of mood disorders and early-onset dementias^34^. Given chronic administration necessary to treat chronic neuroinflammation, significant side effects and/or diminishing efficacy are common with current therapies^35^. Profound anti-neuroinflammatory effects we and others have observed using CBD, combined with evidence of a favorable side-effect profile^36^ suggests it may improve ability to manage this difficult condition.

A potential problem using CBD therapeutically is lack of selectivity. This drug interacts with and modifies activity of multiple cellular targets^37,38^. An additional issue is co-isolation of other potentially bioactive molecules. Purified CBD extracts contain at least traces of other cannabinoids, including CNS-active THC^39^. We have found CBD efficacy is influenced by THC content, underscoring importance of consistent, carefully-controlled formulations^40^. By identifying pathways affected by CBD treatment, it may be possible to identify more selective drugs, reducing potential off-target effects. Alternatively, an entourage of CBD’s diverse cellular interactions may be key to efficacy, and necessary for neuroprotection. The microlesion model shows promise for identifying anti-neuroinflammatory mechanisms and screening potential new drugs.

CBD anti-inflammatory effects appeared greatest in the microlesioned region, HVC, and to progressively lesser extents within RA and Area X (e.g., Fig. 2A–C). This coincides with proximity to HVC, consistent with expected impact on shorter degenerating projections preceding longer ones (note cupric-silver staining evidence for this in supplementary Fig. 7). Because RA integrates output from both the Area X-associated anterior forebrain learning pathway (AFP), and the posterior motor pathway (via HVC, see Fig. 1) we expected disrupted motor input prior to that of the AFP learning circuit^41^. The AFP has an error-generating function that introduces vocal variability critical for sensorimotor learning and modulates activity in both RA^42^ and HVC (indirectly via midbrain dopaminergic nuclei^43^). Persistence of AFP error generation, under conditions of reduced HVC motor control, is consistent with microlesion-induced vocal disruption. This may explain why minimal microlesion effects are observed in AFP-deficient birds^11^.

Another potential explanation for regional differences in anti-inflammatory responsiveness is the timing of these processes that are often coordinated and sequential^44^. Because we investigated only a single 24 h timepoint, we don’t know if responses were just beginning, ending or at peak magnitude.

### CBD anti-oxidant activity

A second mechanism identified included CBD mitigation of oxidative stress, as indicated by effects on SOD2 expression within HVC and RA (Fig. 2E). Oxidative stress effects were further confirmed by reduced superoxide-activated DHE staining^45^ (Fig. 3). Like cytokines, the magnitude of superoxide production varied with lesion proximity, but was significantly decreased by CBD in HVC and RA (Fig. 3B). Note that regional measures of DHE fluorescence are expressed relative to surrounding regions, and therefore demonstrate selective, within song circuit effects.

### CBD promotion of NRF2 signaling

The combination of anti-inflammatory and anti-oxidative CBD activity suggested involvement of a higher order, organized stress response. Consistent with this is signaling controlled by NRF2, an established central regulator of redox, mitochondrial and inflammatory mediators. Under basal conditions NRF2 is a cytoplasmic protein that upon oxidative stress is activated by phosphorylation^17^. Activated phospho-NRF2 translocates to the nucleus where it acts as a transcription factor regulating a host of genes involved in antioxidant, autophagic, misfolded protein and other cellular responses^46^. The significant CBD-related increases in nuclear phospho-NRF2 observed in our system (see Fig. 5) implicates this homeostatic pathway in our model. The fact that CBD effectively both: (1) reduced oxidative stress that typically activates NRF2 and; (2) increased pNRF2 nuclear translocation, suggests CBD anti-oxidant activity is either downstream of pNRF2 activation, or CBD is able to activate NRF2 despite reduced reactive oxygen species (or some combination of both possibilities). Additional experiments investigating time-dependent effects of CBD will be necessary to clarify the nature of NRF2 signaling in this system. Note other activators of NRF2 signaling are clinically-relevant anti-inflammatories^47^. NRF2 is also potently activated by the botanically-derived antioxidant sulforaphane, a derivative of which is currently being evaluated in CNS hemorrhage^48^. These more selective drugs are candidates for anti-neuroinflammatory evaluation in the HVC microlesion system.

### Evidence for CBD effects on microglia

The type of pro-inflammatory cytokine expression we observe following microlesions (described above) is, in other systems, associated with microglial activation, infiltration, and phagocytosis of cellular debris. These activities may be of key importance to neuronal recovery vs. apoptosis^26^. This led us to investigate potential microglia-related activity following HVC microlesions and CBD-improved vocal recovery. Note that this investigation of microglia involvement represents an initial first step. It is highly likely that other cell types are involved in both lesion-induced inflammation and CBD’s anti-inflammatory activity (e.g. reactive astrocytes)^28^. Experiments using additional markers are currently planned and will result in a more complete characterization of cell types active in, and relevant to, our system.

Presently, using TMEM119 as a microglia marker^49^ we found CBD significantly reduced staining densities consistent with reduced myeloid cell infiltration (Fig. 6A,B). This is interesting as it suggests that damage-induced myeloid cell activity, at the single early 24 h timepoint we investigated, is associated with disruptive effects of the microlesions, and not neuroprotection. The rounded cellular appearance of TMEM119-stained cells 24 h post-lesioning suggests, but does not prove, that microlesions increase densities of microglia in an activated phagocytic state, and that CBD treatments reduce this (as shown in Fig. 6Aa-b). Going forward it will be important to investigate a more complete time course of lesion effects. Another caveat follows recent evidence suggesting TMEM119 may not distinguish microglia from migrating peripheral macrophages as reliably as previously thought, and that it does not distinguish activated from inactive states^50^. Because microglia can adopt a continuum of activation states: from pro-inflammatory “M1-like” to anti-inflammatory “M2-like” subtypes^51^ it will be important in future studies to measure multiple markers to distinguish the types and activity of myeloid cell responses^50,52^. Unlike other CBD-related measures, effects on lesion-increased TMEM119 densities were not reversed to unlesioned control levels (Supplemental Fig. S4). A hypothesis we are currently testing is ability of CBD to shift the relative populations of pro- to anti-inflammatory microglial species.

### CBD effects on excitatory synapse densities

A key function of anti-inflammatory microglial subtypes and other myeloid cells^28^ in recovery from CNS damage includes preservation of synaptic densities^53^. Potential CBD protection/promotion of synaptic density was tested by measuring colocalization of PSD95 and the presynaptic glutamatergic marker VGLUT2 (note HVC projections to Area X and RA are glutamatergic^54^). As expected, relative to unlesioned hemispheres, HVC microlesions appeared to decrease densities within the region itself, and also within its projection targets (Figs. 7 and 10b, see also cupric-silver evidence of degeneration [Supplemental Fig. S7]). Less expectedly, CBD increased synaptic marker colocalization within unlesioned hemispheres relative to VEH controls, suggesting promotion of de novo synaptogenesis. Whether synaptogenic activity is accompanied by additional synaptoprotection remains an open question (Figs. 7, 8, 9). The decreased magnitude of vocal disruption seen following CBD treatments suggests potential protection of circuits established during song learning. Facilitating establishment of new synapses may underly CBD promotion of sensorimotor learning-dependent vocal recovery.

### Microlesion and CBD effects on synaptic scaling regulators

A mechanism by which CBD may protect excitatory synapses is through modulating synaptic scaling. This homeostatic process governs synapse sensitivity under various excitation states^55^. Synaptic scaling is regulated by a complex network of proteins and signaling pathways, including BDNF, MSK1 and Arc/Arg3.1^32,56^. BDNF activates MSK1 that in turn acts to alter expression of Arc/Arg3.1^31^. Arc/Arg3.1 directly regulates synaptic localization of excitatory AMPA receptor subtypes in a manner critical for homeostatic protection of learning-related plasticity and memory consolidation^57^. Arc/Arg3.1 activity increases internalization of excitatory AMPA receptors, decreasing and scaling-down excitatory synaptic strength. This regulation may protect against excitotoxicity but, to the extent patterns of AMPA receptor expression are important for maintenance of song circuits established during vocal learning, increased Arc/Arg3.1 may result in the vocal disruption observed in vehicle-treated birds. Decreased magnitudes of vocal disruption observed in CBD-treated birds may be due to reduced synaptic scaling following lesion-related excitotoxity (evident from CuAg staining, Supplemental Fig. S7).

## Conclusions

Taken together, our results demonstrate powerful anti-inflammatory and synaptoprotective mechanisms of CBD action following damage to a pre-motor cortical-like region. This efficacy is associated with promotion of multiple homeostasis-related mechanisms within song circuits. Future studies may link these effects with previously-demonstrated learning-dependent vocal recovery.

## Methods

### Materials

Unless otherwise indicated, all materials and reagents were purchased from Sigma Aldrich or Thermo Fisher. Botanically derived CBD (≥ 98%) was provided by GW Research Ltd, Cambridge, UK. Concentrated stocks of CBD were prepared using nitrogen-sparged 100% ETOH and stored at − 20 °C. Stocks were then diluted with vehicle (2:1:17, ETOH:Alkamuls:PBS) to produce suspensions for injections of 10 mg/kg CBD. Resulting ethanol dosages were 0.33 mg/kg—lower than that voluntarily consumed by zebra finches^58^. Isoflurane (Pivetal, NDC 46,066-755-03) used for anesthesia was provided by the Department of Comparative Medicine at East Carolina University.

### Drug treatments

Stocks of CBD, prepared as described above, were stored in sterile 5 ml septum-capped vials at 4 °C. Fresh stocks were prepared at least weekly. For injections drug preparations were loaded into sterile 1 cc insulin syringe with 30 ga needles. In the morning of injections while the lights were off, birds were captured by hand and the pectoralis muscle injection site was exposed by matting feathers with a small volume of 70% ETOH delivered by squirt bottle. Injections of 50 µl were made into one of four quadrants of pectoralis, rotating daily to minimize potential damage caused by repeated treatments.

### Experimental design

Experiments spanned 8 days. Six daily IM injections of 15 μl were given prior to the surgical procedures to allow CBD, a lipophilic drug with large volume of distribution and long elimination half-life, to approximate steady state levels^59^. On day 7, birds were given a pre-operative injection and unilateral microlesion surgeries were performed according to methods detailed below. Note that unilateral HVC microlesions significantly, but temporarily disrupt vocalizations in a manner consistent with the bilateral approach used previously, except that the magnitude of disruptions was predictably reduced (see Supplemental Fig S1). Maximal behavioral effects of unilateral HVC microlesions were observed at 24 h post-microlesion which is the timepoint used for the present experiments. On day 8, birds were given a post-operative CBD injection in the morning and euthanized in the afternoon for RNA extraction, or perfusion and isolation of paraformaldehyde-fixed brain tissue. All methods were performed in accordance with the relevant guidelines and regulations.

### Animals and environment

Adult male zebra finches (> 90 days of age) were raised in our breeding aviary and maintained at 78 °F on a 12/12 light/dark cycle. Males were exclusively used due to their ability to produce song. Birds were housed in standard finch cages (9″ × 11″ × 17″) with ad libitum food and water. Birds were visually but not auditorily isolated, consistent with prior experiments done in recording chambers^12^. All animal procedures were approved by the East Carolina University Animal Care and Use Committee (see ethics declarations below) and this study was conducted and reported in accordance with the ARRIVE guidelines^60^.

### HVC microlesion surgeries

To reduce animal impact and improve statistical power we modified our original bilateral microlesion model^12^ to adopt a unilateral approach that allows individual subjects to serve as their own internal control. We targeted left hemispheres as evidence indicates lateralization similar to that characteristic of human language^61^ further illustrating parallels between speech and birdsong (also, initial optimization experiments suggested left hemisphere HVC microlesion disrupted vocal quality to a greater degree than those targeting right hemispheres, see Supplemental Fig S1). With exception of the bilateral modification, microlesions were done as described previously^12^. Briefly, using a MIDMARK VMS anesthesia system (91,800,003 VMS), birds were initially anaesthetized using 3% isoflurane (Vaporized with an oxygen carrier at a constant flow rate of 1.0 L/min). Feathers from the top and back of the head were removed and birds were secured in a stereotaxic instrument with two metal rods inserted into the ear canal and beak placed on beak bar. Affixed to the beak bar was a 20-gauge tube for constant delivery of isoflurane during the procedure. The bifurcation at the midsagittal sinus was used as stereotaxic zero. Small craniotomies were placed over the left HVC (after skull section removal, isoflurane was lowered to 2%). For approximately 8% destruction of left HVC, two locations were targeted: 2.4 and 2.8 mm from the stereotaxic zero to a depth of 0.6 mm. Microlesions were made with 100 µA for 35 s. After lesioning, isoflurane was lowered to 1% before suturing. Birds recovered in a warm incubator and were returned to recording chambers. Note these methods were adapted from those originally developed by Thompson and Johnson, 2007^11^. Also note that our prior study^12^ demonstrating CBD efficacy to improve vocal recovery employed a bilateral lesion model, and not the unilateral approach adopted here. This is a limitation as we do not demonstrate CBD efficacy to improve vocal recovery following our revised, unlateral lesion method. Therefore, mechanisms we identify as associated with CBD efficacy are not causally linked to prior demonstration of improved vocal recovery.

### Quantitative RT-PCR

Gene expression experiments were performed with three biological replicates from four to five adult zebra finches per treatment group. For each animal, a sterile RNAse free 1 mm diameter biopsy punch tool was used to excise brain tissue of three regions of interest, from each hemisphere (Unlesioned right used as an internal control and lesioned left): HVC; RA; and Area X. Example images are shown in Supplemental Fig. S2. Brain samples were homogenized in TRIzol reagent (Invitrogen, 15,596,026) separated using chloroform and precipitated using isopropanol. Precipitated RNA was washed and resuspended in RNase free water. RNA quality was confirmed by gel electrophoresis. Total RNA (250 ng) was used to synthesize cDNA using an iScript synthesis kit (Bio-rad, 1,708,890). Completed reactions were diluted fivefold, in triplicate using RNAse free water. PCR was done using SYBR green supermix (Bio-Rad, 1,725,271). Selective amplification was confirmed using a melt curve analysis and data were obtained as cycle threshold (Ct) values using CFX Manager software (Bio-Rad). Gene expression was normalized to the endogenous control (GAPDH) and fold change was determined from the unlesioned hemisphere using the ΔΔCt method^62^. Primer sequences and information are located in Supplemental Table S1.

### Fixation and cryosectioning

Adult male zebra finches (n = 3–5) were administered drug treatments for 6 days, microlesioned on day 7 and transcardial perfusions were performed 24 h later using 4% paraformaldehyde for fixation and 30% sucrose for cryoprotection. Fixed brains were blocked at the midline, placed in OCT embedding medium, frozen using a slurry of 2-methyl butane and dry ice, then sectioned at 10 µm using a cryostat kept at − 20 °C. Parasagittal sections of both right and left hemispheres from each bird were mounted on Superfrost Plus slides and stored at − 20 °C.

### Immunofluorescent staining

Slides were blocked with 5% normal goat serum for 1 h at 37 °C. Primary antibodies targeting various proteins were diluted in 2% normal goat serum and used at optimized concentrations: anti-IL-6, 10 µg/ml (Biomatik, CAU30440); anti-IL-1B, 10 µg/ml (Mybiosource, MBS 2,090,494); anti-IL-10, 1:100 (BIOSS, AGO7251283); anti-PSD95, 1:50 (Santa Cruz, SC-32291); anti-vesicular GLUT2 (VGLUT2) 1:500 (Cell Signaling, 715,555); anti-TMEM119, 1:100 (Abcam, AB185337); anti-phosphorylated NRF2 (pNRF2), 1:200 (Abcam, AB76026); and nuclear counterstain Hoechst, 1:10,000 (Thermo Fisher Scientific, H3570). Primary antibody specificity were validated via western blotting, images of which are summarized in Supplemental Fig S6. All primary antibodies were incubated at 4 °C overnight. The following day, slides were washed with PBS then was incubated in corresponding secondary antibody diluted in 2% normal goat serum at 37 °C for 1 h. Secondary antibodies were Alexa Fluor 488 goat anti-mouse (A32723) Alexa Fluor 647 goat anti-mouse (A32728) Alexa Fluor 647 goat anti-rabbit (A32733). After incubation of one hour in secondary antibody, slides were washed twice with PBS for five minutes each followed by Hoechst nuclear counterstain. After fourth and final wash, cover slips were placed on slides using diamond antifade mountant (Invitrogen, P36961). Control reactions were done without primary antibodies to demonstrate lack of significant nonspecific binding of secondary antibodies (see Fig S8).

### Western blotting

Western blot analyses were done to determine specificity of primary antibodies used with zebra finch tissue (see supplementary Fig. 6). For each blot, protein samples were prepared from homogenized brain tissue (Fisher Scientific, PowerGen 1000 S1) in RIPA lysis and Extraction Buffer (Thermo, 89,900) with protease inhibitor (Thermo, A32953). After homogenization, mixture was agitated on a Rotomix for 2 h at 4 °C. Then tissue was centrifuged at 16,000 *g* for 20 min at 4 °C, pellet was discarded and supernatant collected. Lysis buffer with protease inhibitor was made fresh for each extraction. Protein concentration was determined by Pierce BCA assay (Thermo Fisher Scientific, 23,227) using lysis buffer as diluent for assay and subsequent dilutions.

For electrophoretic separation, 4 µL of precision plus dual color protein ladder (Bio-Rad, #1,610,374) was loaded into lane 1 of a 10% Mini- PROTEAN TGX gel (Bio-Rad, #4,561,034) followed by 20 µg of zebra finch brain protein (denatured with 4 × laemmli loading buffer at 95 °C for 5 min) in lane 2. Electrophoresis was run at a constant 100 V for 75 min. Protein was transferred onto methanol activated PVDF membrane using a Trans-Blot Turbo System (Bio-Rad, 1,704,150) with parameters determined by respective molecular weight. Membrane was then blocked in filtered 5% BSA in 0.1% TBST for 3–4 h at 37 °C. Blocking buffer was removed, 7 ml primary antibody diluted in 0.05% TBST added, and then was incubated on a rocker in 4 °C overnight. Antibodies were optimized and concentrations used were as follows: anti-IL-6, 10 µg/ml (Biomatik, CAU30440); anti-IL-1B, 2 µg/ml (Mybiosource, MBS 2,090,494); Anti-IL-10, 1:1000 (BIOSS, bs-0698R-TR); anti-PSD95, 1:100 (Santa Cruz, SC-32291); anti-vesicular GLUT2 (VGLUT2) 1:1000 (Cell Signaling, 715,555); anti-TMEM119, 1:200 (Abcam, AB185337); anti-phosphorylated NRF2 (pNRF2), 1:1000 (Abcam, AB76026). All dilution buffers were prepared fresh prior to use. The following day, primary antibody was removed and membrane washed 4 times with 0.1% TBST for 10 min each. After the 4th wash, membranes were incubated in appropriate secondary antibody for 1 h on a rocker at 37 °C. Secondary antibodies were IRdye 680RD Goat anti-rabbit 1:10,000 (Licor, 925–68,071) and IRdye 800CW Goat anti-mouse 1:10,000 (Licor, 925–32,210). After incubation, secondary antibody was removed and membrane was wash 4 times with 0.1% TBST for 10 min each. After washes, images were captured using an Odyssey M Imager (Licor, M3350).

### Dihydroethidium (DHE) staining

Superoxide anions were detected via 5 µM Dihydroethidium (DHE, Invitrogen, D11347) following a previously described protocol^63^. DHE freely permeates cell membranes and reacts with cytosolic superoxide (O_2_^−^) producing ethidium that fluoresces red upon DNA binding. This fluorescence can then be quantified^64^. Briefly, brain tissue was rapidly dissected and flash frozen in OCT compound using a dry ice 2-methylbutane slurry. Using a freezing microtome (Epredia Microm HM525 NX Cryostat) 10 µm sections were cut and mounted on Fisher Superfrost Plus microscope slides and then 1 mL of 5 µM DHE diluted in PBS was gently pipetted onto each slide and incubated at 37 °C for 30 min protected from light, rinsed two times with PBS, and imaged.

### Dark-field and confocal imaging

Dark-field images for regional identification were obtained using Image-Pro Plus software (version 6.3) and an Olympus BX51 microscope equipped with a darkfield condenser at 12.5X (Supplemental Fig. S2). Borders of regions of interest were traced from darkfield images for later superimposition over fluorescent confocal images. Regions of interest included HVC outside of infarcts, RA, and Area X. Sections that contained portions of all three regions of interest were identified to ensure equal representation across treatment conditions. Fluorescent images were obtained using a Zeiss laser scanning microscope (LSM, 700 Axio Observer) with 40X (Plan-Apochromat/1.4 Oil DIC M27) and 10X objectives (EC Plan-Neofluar/0.30 M27). Using Zeiss ZEN Black imaging software, Z-stack images were compiled using 5 slices and analyzed after superimposing at maximum intensity using Image J.

### Image analysis

Image J software was used to analyze superimposed z-stack images converted to 8-bit with a threshold applied consistently across all images within each region. All CZI-format image files were exported from ZEN Black software as greyscale tiff files and consolidated into a maximum intensity z projection. For IL-6, IL-1B, and IL-10, mean grey value of individual stain relative to Hoechst nuclear stain by song region was quantified as a percentage of the control hemisphere. Note that raw, untransformed relative fluorescence measures of cytokine expression for both lesioned and unlesioned hemispheres hemisphere are summarized in Supplemental Fig. S5. For DHE, mean grey values were determined and corrected for background fluorescence using the average intensity outside of each song region studied (Fig. 3B). Groups were compared using a percentage of the control hemisphere (Fig. 3C). Mean corrected total cell fluorescence (CTCF) was used to eliminate background using a consistent circular area (circular diameter of 0.5 mm): CTCF = Integrated Density—(mean song region intensity * mean background intensity). For analysis of nuclear localization of pNRF2, color thresholding was used to determine: (1) the area of pNRF2 colocalized with Hoechst nuclear staining and (2) the total area of Hoechst nuclear staining. pNRF2 nuclear area was then expressed as a percentage of total nuclear area and compared across groups (Fig. 4B). For analysis of the microglia marker TMEM119 within each region, mean grey value of TMEM119 staining was normalized to Hoechst-stained nuclei and calculated as percent change from unlesioned control hemisphere (Fig. 6B). Lastly, glutamatergic synapse densities were determined using the colocalization of VGLUT2 and PSD95 per area of measurement (circular diameter of 100 µm).

### Ethical approval

Animals were used following protocols approved by the Institutional Animal Care and Use Committee at East Carolina University (ECU-IACUC). ECU-IACUC oversees a registered research facility under the Animal Welfare Act (#55-R-0010) and has an approved Animal Welfare Assurance Statement with the Office of Laboratory Animal Welfare D16-00,294. In addition, ECU has continued full accreditation by the Association for Assessment and Accreditation of Laboratory Animal Care (AAALAC). This work involving animals, specifically adult male zebra finches, was confirmed to follow all ARRIVE guidelines^60^ including but not limited to planning the experimentation and conducting the approved research, as well as writing and reviewing the manuscript to ensure all information is available and easily accessible.

### Statistical analysis

Statistical analyses were performed with GraphPad Prism 9.2.0 for all histological, behavioral, and gene expression data. Data are expressed as mean ± SEM. Statistical analysis was performed using a mixed-models ANOVA followed by Sidak’s post hoc analysis to identify differences between groups. A *p* value ≤ 0.05 was considered statistically significant. Statistical results are indicated in the text and figure legends.

## Supplementary Information

Supplementary Information.

## Data Availability

The datasets used during the current study is available from the corresponding author upon reasonable request.
