# Supplementary material for: Cannabidiol inhibits neuroinflammatory responses and circuit-associated synaptic loss following damage to a songbird vocal pre-motor cortical-like region
Source: Sci Rep. 2023 May 16;13:7907. doi: 10.1038/s41598-023-34924-z (PMC10188539; doi:10.1038/s41598-023-34924-z)
Supplement: Supplementary file 1 — Supplementary Information. [file 41598_2023_34924_MOESM1_ESM.pdf]

CANNABIDIOL INHIBITS NEUROINFLAMMATORY RESPONSES AND CIRCUIT-ASSOCIATED SYNAPTIC LOSS FOLLOWING DAMAGE TO A SONGBIRD VOCAL PRE-MOTOR CORTICAL-LIKE REGION

Mark Tripson, Karen Litwa, and Ken Soderstrom

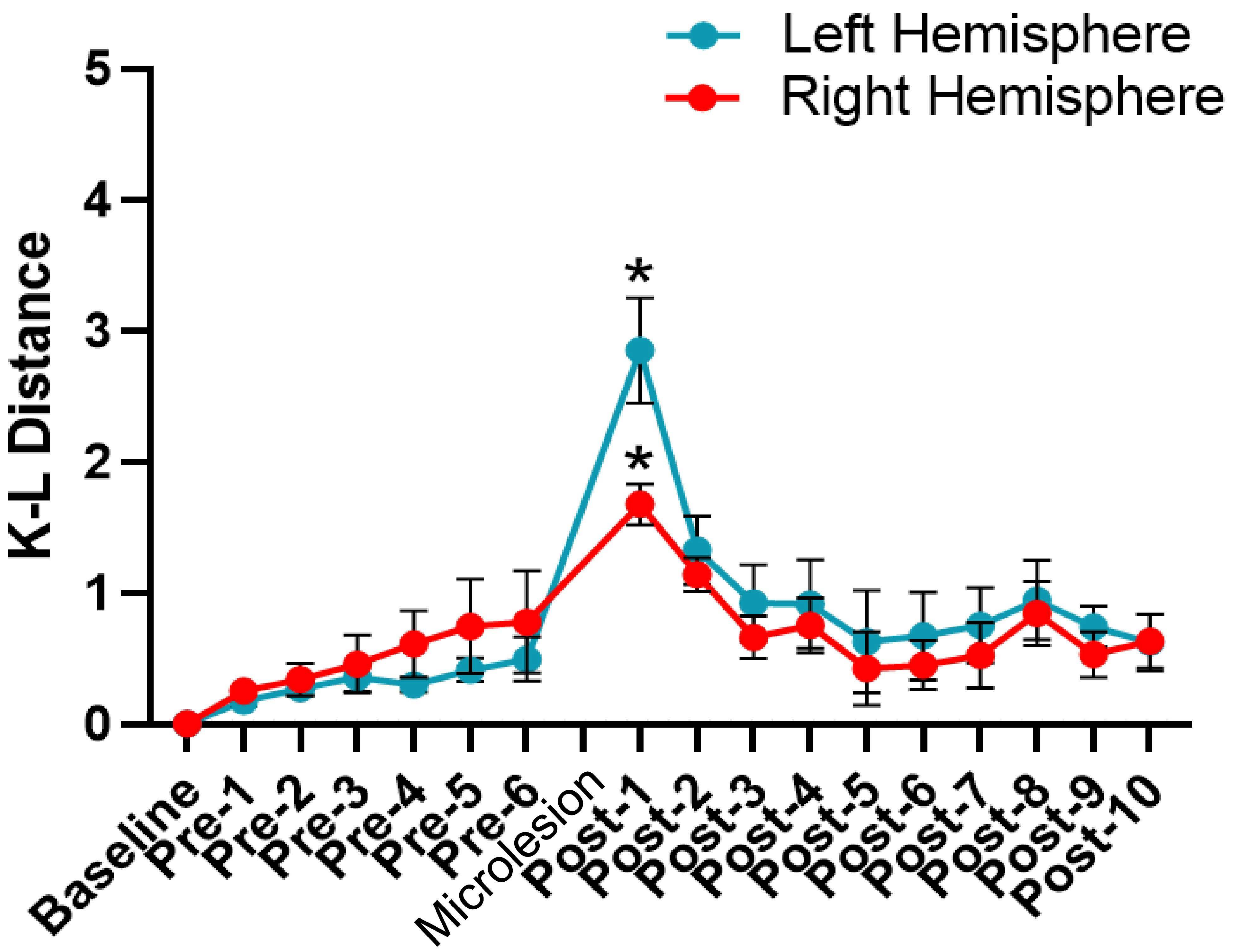
Left Hemisphere
Right Hemisphere
K-L Distance
0
1
2
3
4
5
*
*
Baseline
Pre-1
Pre-2
Pre-3
Pre-4
Pre-5
Pre-6
Microlesion
Post-1
Post-2
Post-3
Post-4
Post-5
Post-6
Post-7
Post-8
Post-9
Post-10

Supplemental Figure 1. Unilateral HVC microlesions temporarily disrupt zebra finch song. Y-axis are days relative to microlesions given on day 7. Using a measure of vocal variability, K-L distance[13,64], a unilateral HVC microlesion to either the right or left hemisphere, disrupts vocalization peaking 24 hours after surgery. Interestingly, left hemisphere lesions produced a greater change from baseline, although both were statistically significant. Birds were recorded continuously for the entire 20 day experimental period. Daily recordings from n = 3 birds/group were acoustically-analyzed and KL distance measures generated as previously described[1].

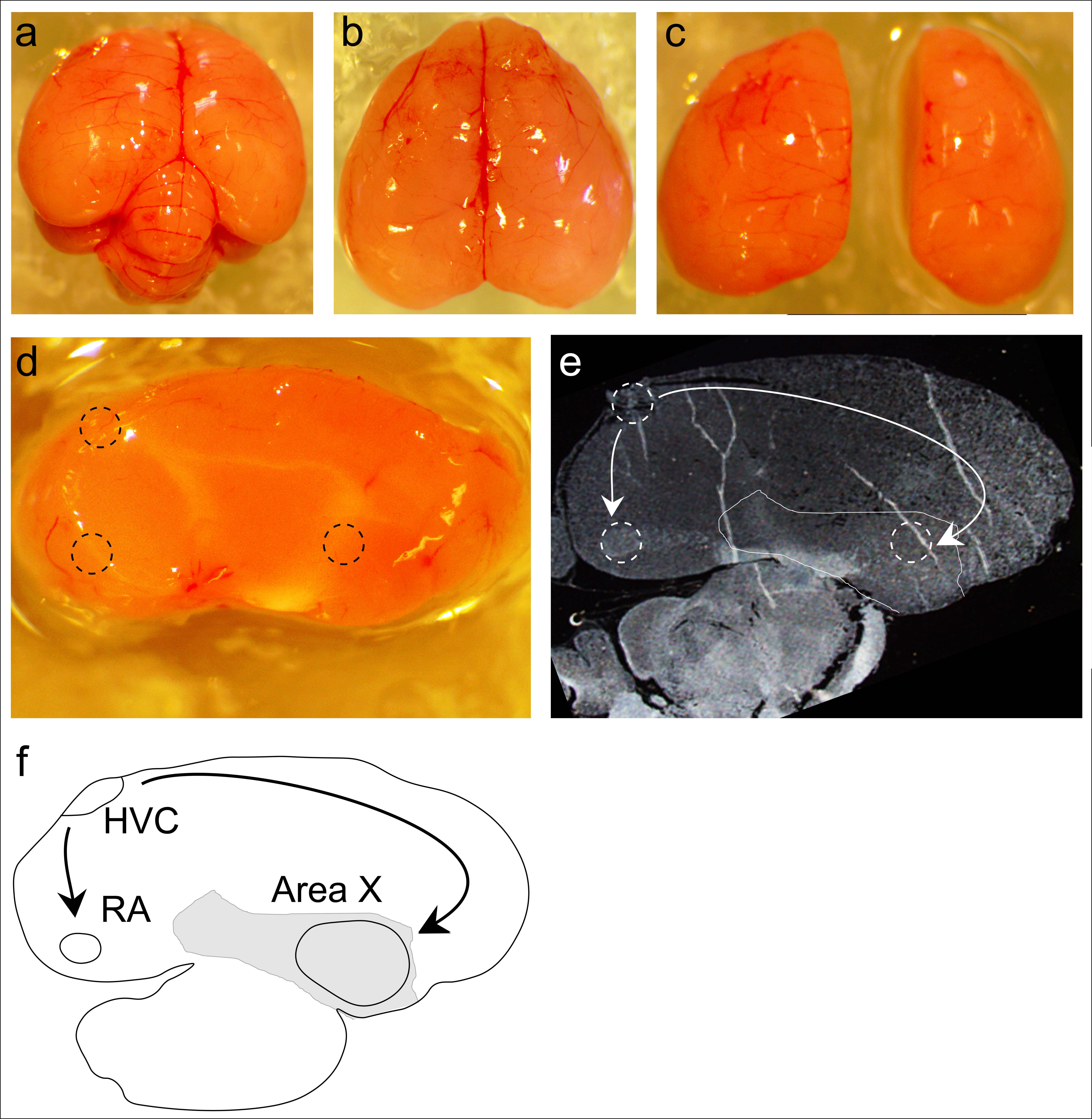
a
b
c
d
e
f
HVC
RA
Area X

Supplemental Figure 2. Illustration of micro punch technique used to isolate regions of interest. A-D, Gross dissection of fresh zebra finch brain tissue and biopsy punch locations based on fiber tracts in a sagittal section. E, Dark-field image used to identify tissue sections with all regions of interest. Dense regions are circled, and arrows indicate circuits that connect them. F, Outline of sagittal section with areas of interest highlighted. Rostral is approximately right, dorsal up, and bar = 1 mm.

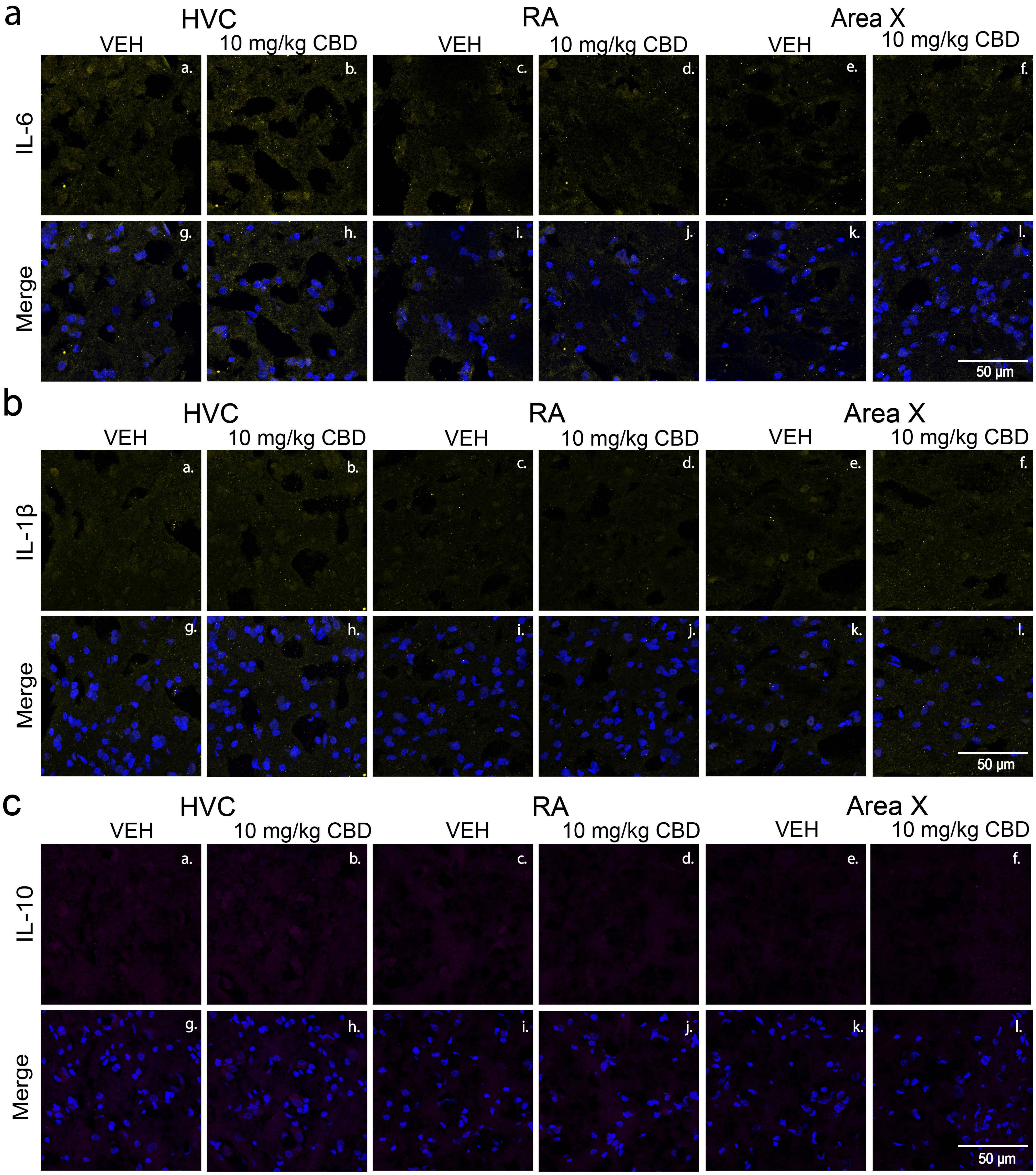
a
HVC
RA
Area X
VEH
10 mg/kg CBD
IL-6
Merge
a.
b.
c.
d.
e.
f.
g.
h.
i.
j.
k.
l.
50 μm
b
IL-1β
Merge
50 μm
c
IL-10
Merge
50 μm

Supplemental Figure 3. Representative immunofluorescent confocal images of antibodies targeting IL-1B, IL-6 and IL-10 within motor (HVC & RA) and learning-essential (Area X) regions of contralateral (unlesioned) hemispheres. Note that little staining is observed. Aa-Al, Unlesioned hemisphere images showing representative regional distribution of IL-6 in vehicle- vs. CBD-treated birds. Ba-Bl, images demonstrating regional distribution of IL-1B. Ca-Cl, confocal images demonstrating regional distribution of IL-10.

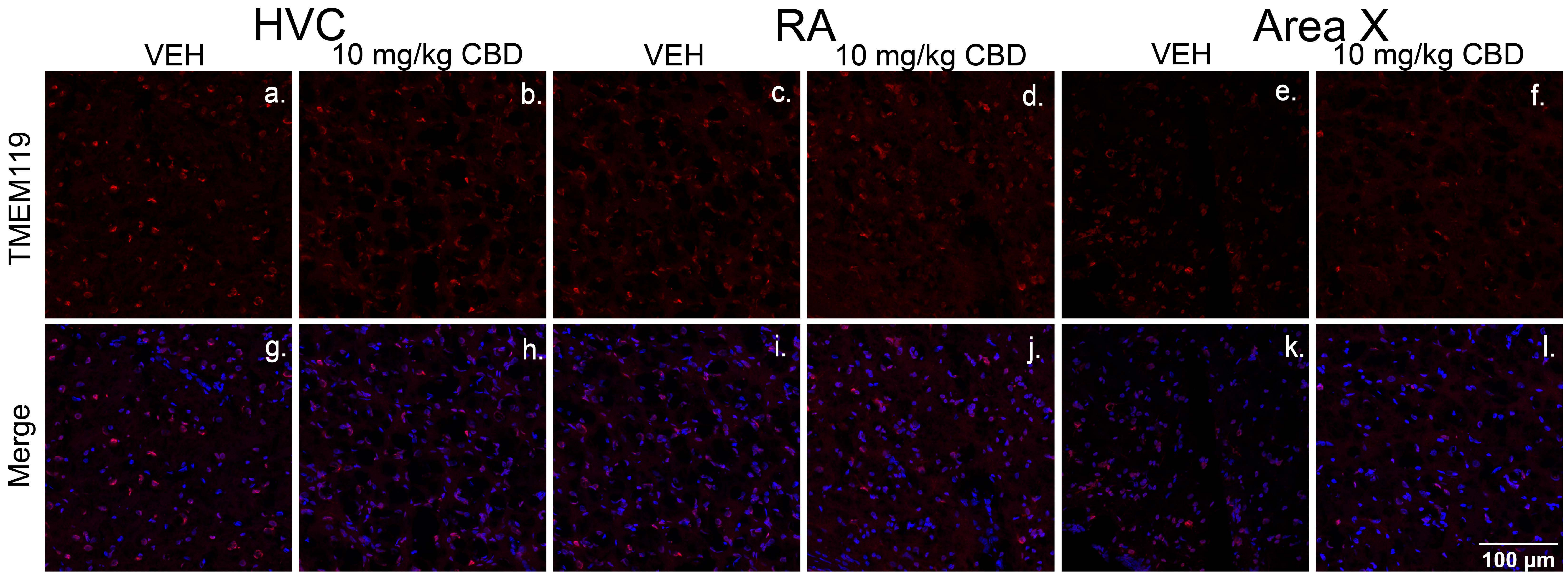

HVC
RA
Area X
VEH
10 mg/kg CBD
VEH
10 mg/kg CBD
VEH
10 mg/kg CBD
TMEM119
Merge
a.
b.
c.
d.
e.
f.
g.
h.
i.
j.
k.
l.
100 µm

Supplemental Figure 4. Representative immunofluorescent confocal images of antibodies targeting TMEM119 within motor (HVC & RA) and learning-essential (Area X) regions of the contralateral (unlesioned) hemispheres. Aa – Af, TMEM119 immunofluorescence marks microglia. Ag – Al, Merge images of TMEM119 and Hoechst-stained nuclei.

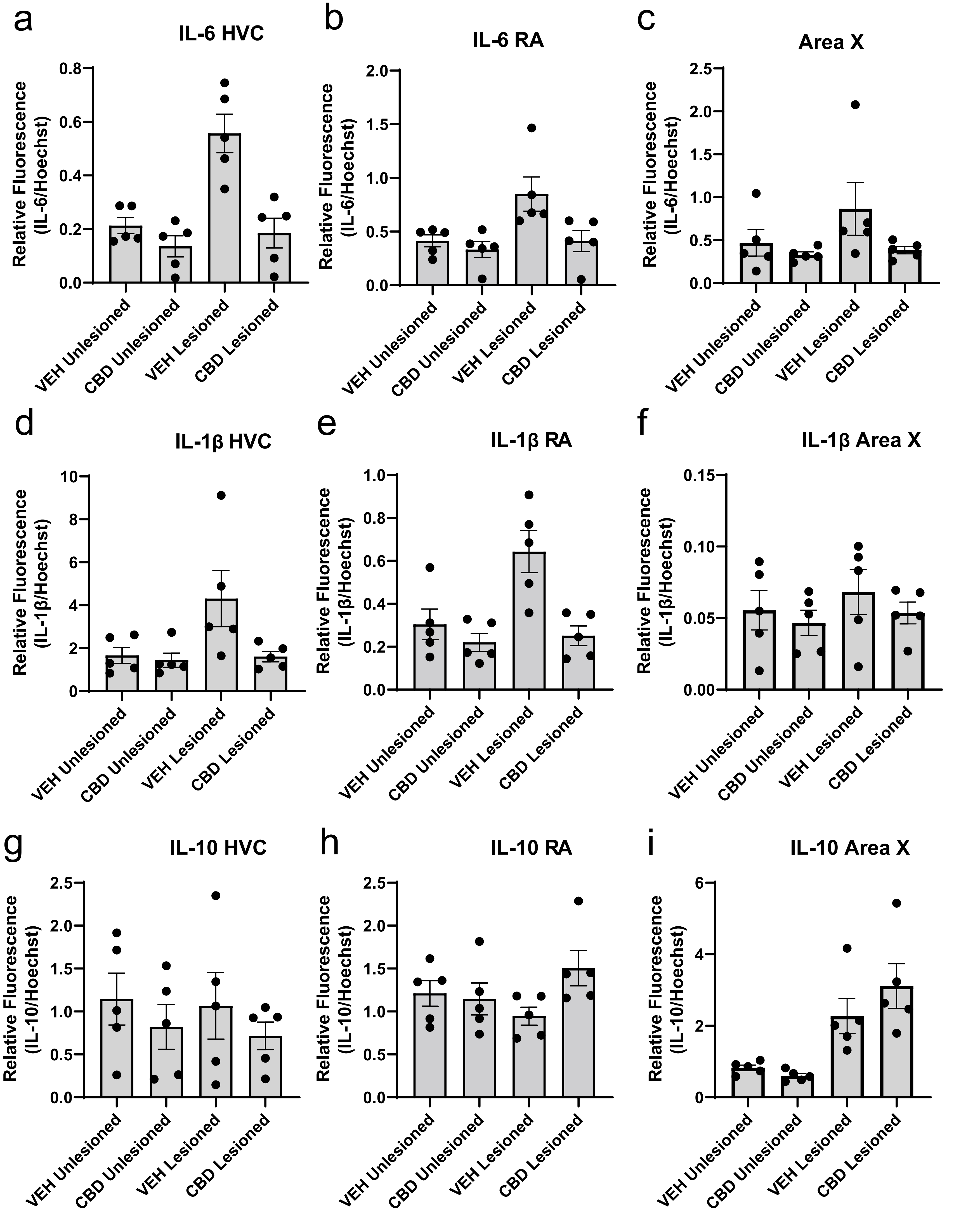

a
IL-6 HVC
Relative Fluorescence (IL-6/Hoechst)
0.0
0.2
0.4
0.6
0.8
VEH Unlesioned
CBD Unlesioned
VEH Lesioned
CBD Lesioned
b
IL-6 RA
Relative Fluorescence (IL-6/Hoechst)
0.0
0.5
1.0
1.5
2.0
c
Area X
Relative Fluorescence (IL-6/Hoechst)
0.0
0.5
1.0
1.5
2.0
2.5
d
IL-1β HVC
Relative Fluorescence (IL-1β/Hoechst)
0
2
4
6
8
10
e
IL-1β RA
Relative Fluorescence (IL-1β/Hoechst)
0.0
0.2
0.4
0.6
0.8
1.0
f
IL-1β Area X
Relative Fluorescence (IL-1β/Hoechst)
0.00
0.05
0.10
0.15
g
IL-10 HVC
Relative Fluorescence (IL-10/Hoechst)
0.0
0.5
1.0
1.5
2.0
2.5
h
IL-10 RA
Relative Fluorescence (IL-10/Hoechst)
0.0
0.5
1.0
1.5
2.0
2.5
i
IL-10 Area X
Relative Fluorescence (IL-10/Hoechst)
0
2
4
6

Supplemental Figure 5. Quantification of relative mean grey value of pro- and anti-inflammatory cytokine protein expression. A-C, Relative fluorescence of IL-6 by song region and drug treatment. D-F, Relative fluorescence of IL-1B by song region and drug treatment. G-I, Relative fluorescence of IL-10 by song region and drug treatment.

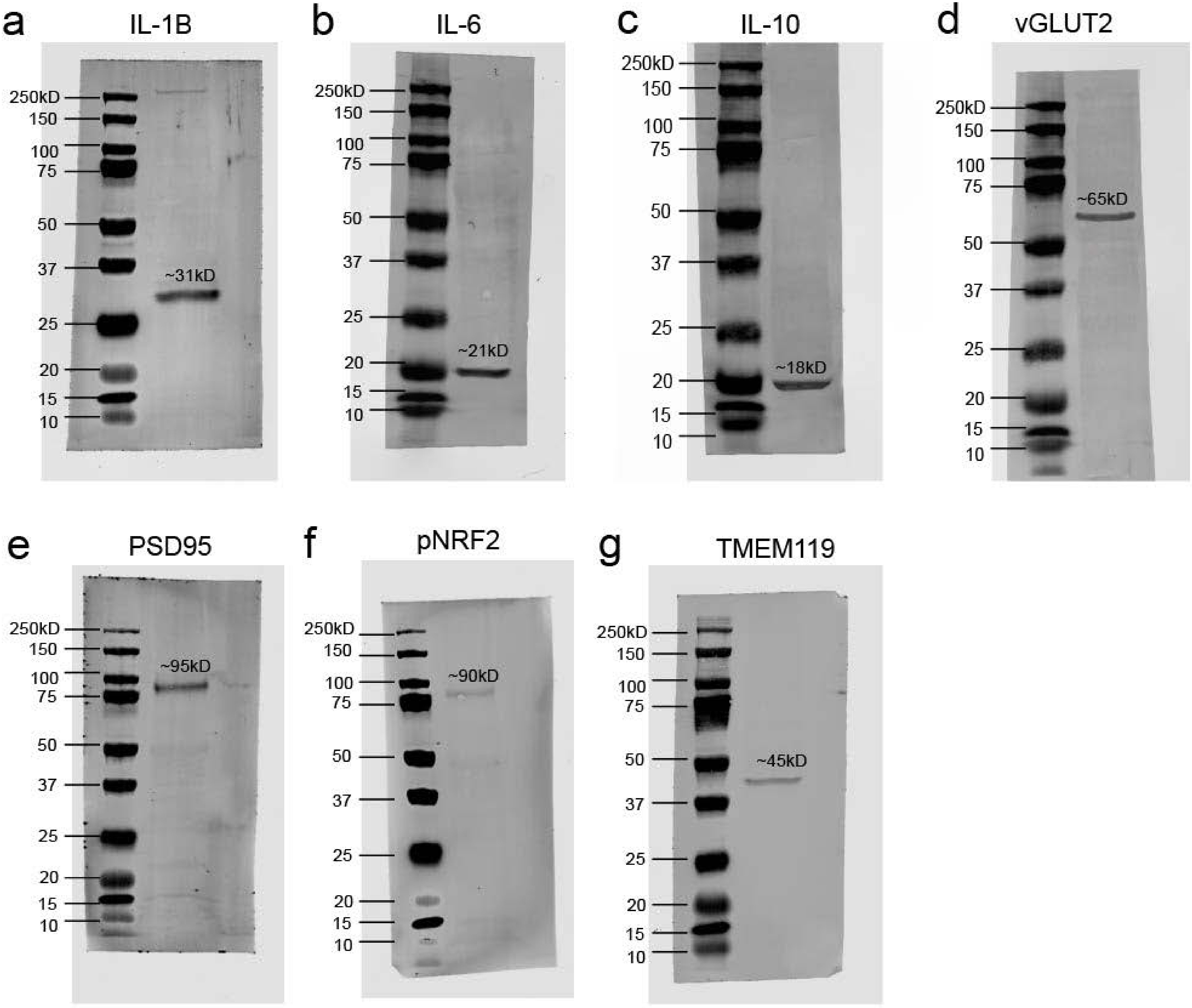

a
IL-1B
250kD
150
100
75
50
37
25
20
15
10
~31kD
b
IL-6
250kD
150
100
75
50
37
25
20
15
10
~21kD
c
IL-10
250kD
150
100
75
50
37
25
20
15
10
~18kD
d
vGLUT2
250kD
150
100
75
50
37
25
20
15
10
~65kD
e
PSD95
250kD
150
100
75
50
37
25
20
15
10
~95kD
f
pNRF2
250kD
150
100
75
50
37
25
20
15
10
~90kD
g
TMEM119
250kD
150
100
75
50
37
25
20
15
10
~45kD

Supplemental Figure 6. Western blotting supports antibody selectivity with zebra finch protein. Each gel was run with ladder and protein. Each antibody was run on different gels along with its respective ladder.

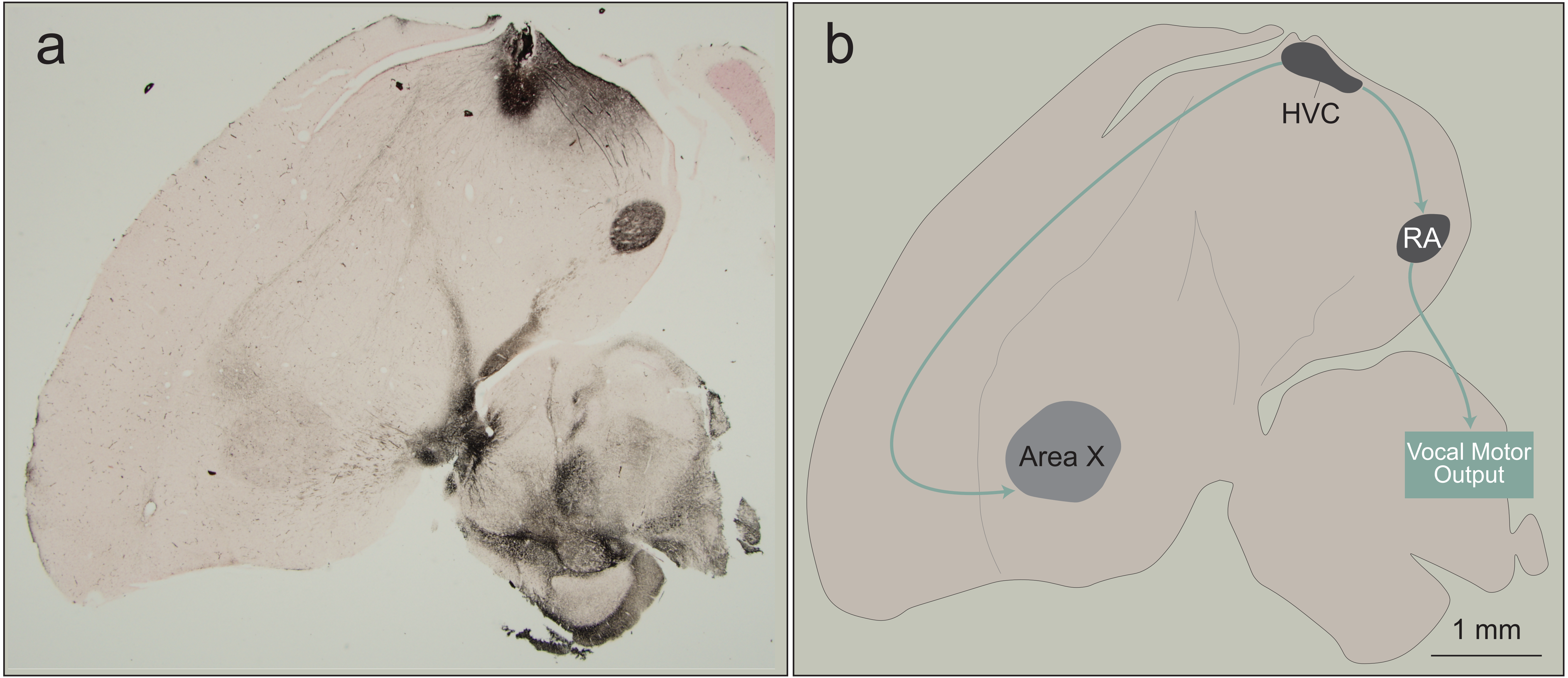
a
b
HVC
RA
Area X
Vocal Motor
Output
1 mm

Supplemental Figure 7. Amino-cupric-silver (A-Cu-Ag) staining to detect neuronal degeneration in zebra finch brain tissue 24 hours after unilateral microlesion (left is anterior, top is dorsal). A, representative image showing microlesion ablation of HVC, significant darkening of projected region RA and slight darkening of Area X. Note, there is axonal degradation from HVC to RA. B, outline of tissue with regions of interest labeled (HVC, RA, and Area X).

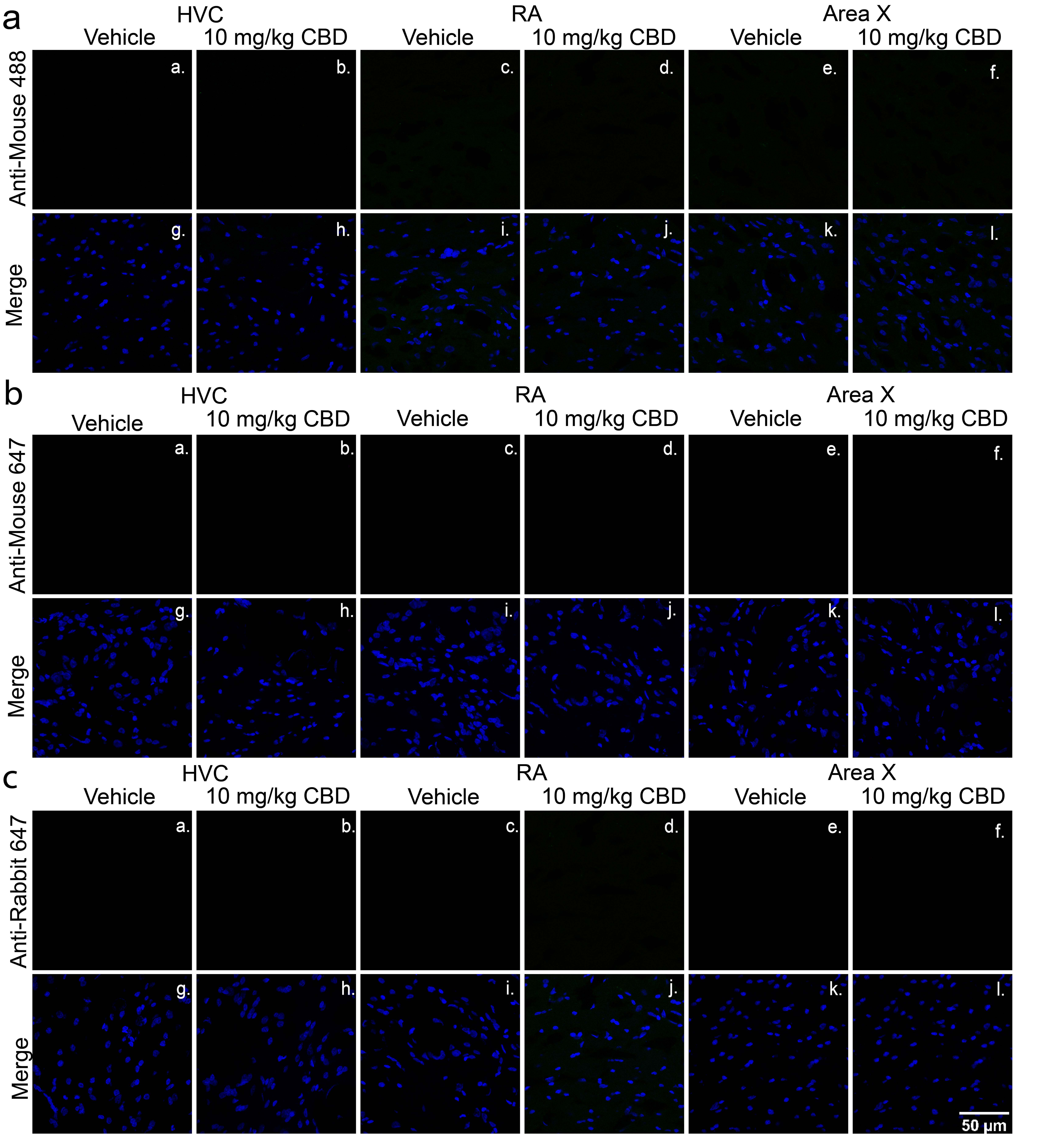
a
HVC
RA
Area X
Vehicle
10 mg/kg CBD
Anti-Mouse 488
Merge
b
Anti-Mouse 647
c
Anti-Rabbit 647
a. b. c. d. e. f. g. h. i. j. k. l.
50 µm

Supplemental Figure 8. No primary controls for immunofluorescent staining. The samples followed the same immunofluorescence staining protocol but were incubated with only the diluent (no primary antibody) and then with the respective secondary antibody. Images were taken using the same LSM700 settings as images taken with primary antibodies.

Gene Symbol	Accession Number	Forward Primer (5’-3’)	Reverse Primer
GAPDH	NM_OO1198610.1	TGCTGCTCAGAACATTATCCC	TTTCCCACAGCCTAGCAGCT
IL-1β	XM_002195564.2	TTCCGGTGCATCAGAGGCAGTTAT	GCACGAAGCACTTGTGGTCAATGT
IL-6	XM_002191284.2	CGTCTGCCAGAACAGCATGGAAAT	TATCCTCATTAAAGCCGGCGAGCA
IL-10	XM_002194605.4	CAAGCTCAAAGAGCTGAGGC	GCCCTTGATTTCCTCCAGCA
TNFα	XM_002197321.2	TGTCCCATCTGC ACCACCTTCTTA	ATTCCCTTCCATCTGGCTTCTGT
SOD2	NM_001245469.2	ACAGGGGCGCCTACAGATAG	CATGTTCCCATACGTCAATGCC
BDNF	XM_012573739.4	TCCAGCATCTGTTGGAGAGAC	CGAAGACCTGGGTAAGCCAA
RPS6KA5 (MSK1)	XM_030274847.3	CGGAGTGCCCCTGGAAAATA	GGCCGCTTACTTTCCTCACT
Arc/Arg3.1	XM_002186825.6	TGGAAGAAGTCCATCAAGGC	TTGCGCCAGAGGAACTGGTC

Supplemental Table 1. Table listing gene symbol, accession number, and forward and reverse primers used for quantitative RT-PCR.

References

1. Alalawi, A. *et al.* Cannabidiol improves vocal learning-dependent recovery from, and reduces magnitude of deficits following, damage to a cortical-like brain region in a songbird pre-clinical animal model. *Neuropharmacology* **158**, 107716 (2019).

2. Soderstrom, K. & Alalawi, A. Software for objective comparison of vocal acoustic features over weeks of audio recording: KLFromRecordingDays. *SoftwareX* **6**, 271–277 (2017).
